# LINC01123, a c-Myc-activated long non-coding RNA, promotes proliferation and aerobic glycolysis of non-small cell lung cancer through miR-199a-5p/c-Myc axis

**DOI:** 10.1186/s13045-019-0773-y

**Published:** 2019-09-05

**Authors:** Qian Hua, Mingming Jin, Baoming Mi, Fei Xu, Tian Li, Li Zhao, Jianjun Liu, Gang Huang

**Affiliations:** 1 Department of Nuclear Medicine, Renji Hospital, School of Medicine, Shanghai Jiaotong University, Shanghai, 200127 China; 2 Shanghai Key Laboratory of Molecular Imaging, Shanghai University of Medicine and Health Sciences, Shanghai, 201318 China; 3 Department of Nuclear Medicine, Affiliated Hospital of Jiangnan University (Wuxi 4th People’s Hospital), Wuxi, 214062 Jiangsu China

**Keywords:** Long non-coding RNAs, LINC01123, non-small cell lung cancer, c-Myc, aerobic glycolysis

## Abstract

**Background:**

Long non-coding RNAs (lncRNAs) have been associated with non-small cell lung cancer (NSCLC), but the underlying molecular mechanisms of their specific roles in mediating aerobic glycolysis have been poorly explored.

**Methods:**

Next-generation RNA sequencing assay was performed to identify the differentially expressed RNAs between NSCLC tissues with high ^18^F-fluorodeoxyglucose (FDG) uptake and their adjacent normal lung tissues. LINC01123 expression in NSCLC tissues was measured by real-time PCR and in situ hybridization (ISH) assay. The biological role of LINC01123 in cell growth and aerobic glycolysis capability was determined by performing functional experiments in vitro and in vivo. Further, the transcription of LINC01123 was explored by bioinformatics analysis, dual-luciferase reporter assay, and chromatin immunoprecipitation (ChIP) assay. RNA immunoprecipitation (RIP) and luciferase analyses were used to confirm the predicted competitive endogenous RNA (ceRNA) mechanisms between LINC01123 and c-Myc.

**Results:**

Three hundred sixty-four differentially expressed genes were identified in RNA-seq assay, and LINC01123 was one of the most overexpressed lncRNAs. Further validation in expanded NSCLC cohorts confirmed that LINC01123 was upregulated in 92 paired NSCLC tissues and associated with poor survival. Functional assays showed that LINC01123 promoted NSCLC cell proliferation and aerobic glycolysis. Mechanistic investigations revealed that LINC01123 was a direct transcriptional target of c-Myc. Meanwhile, LINC01123 increased c-Myc mRNA expression by sponging miR-199a-5p. In addition, rescue experiments showed that LINC01123 functioned as an oncogene depending on miR-199a-5p and c-Myc.

**Conclusion:**

Since LINC01123 is upregulated in NSCLC, correlates with prognosis, and controls proliferation and aerobic glycolysis by a positive feedback loop with c-Myc, it is expected to be a potential biomarker and therapeutic target for NSCLC.

**Electronic supplementary material:**

The online version of this article (10.1186/s13045-019-0773-y) contains supplementary material, which is available to authorized users.

## Introduction

According to the latest annual global cancer statistics report, lung cancer is the most commonly diagnosed cancer (11.6% of the total cases) and the leading cause of cancer death (18.4% of the total cancer deaths) in the whole world [1]. Primary lung cancers are usually classified into non-small cell lung cancer (NSCLC) and small cell lung cancer (SCLC). NSCLC accounts for approximately 83% of all lung cancers, and nearly 80% of NSCLC patients are diagnosed with advanced or distant stages [2]. NSCLC comprises multiple histological, genetical, and metabolical procedures. One predominant feature of NSCLC is the active glucose metabolism, which can be visualized by high ^18^F-FDG uptake on positron emission tomography/computed tomography (PET/CT) imaging [3, 4]. Recently, ^18^F-FDG PET/CT has been recommended routinely used in the clinical staging of NSCLC according to the 8th edition of the TNM staging system [5].

The underlying mechanism of ^18^F-FDG uptake is that cancer cells exhibit aberrant metabolism characterized by high glycolysis even in the presence of abundant oxygen. This metabolic reprogramming, known as the Warburg effect or aerobic glycolysis, has been regarded as a new hallmark of cancer [6, 7]. The Warburg effect facilitates cancer cells to rewire their metabolism to harness cellular stress to thrive and is an optimized way to favor their survival, progression, and metastasis [8]. The aerobic glycolysis of cancer cells is regulated by several master transcription factors, most notably the c-Myc transcription factor [9]. Recent studies have demonstrated that c-Myc, which is a frequently amplified human oncogene, functions as a key regulator of the Warburg effect by directly activating several glycolytic genes [9–11]. Although the underlying mechanisms of the Warburg effect by protein-coding genes have been extensively studied, the potential functions and mechanism of the more recently identified lncRNAs in cancer metabolism remain largely unknown [12].

With the development of RNA sequencing techniques, integrative genomic studies have shown that more than 90% of the DNA sequence is actively transcribed, but only < 2% of these transcripts encode protein, while the majority of the transcripts are referred to as non-coding RNAs (ncRNAs) [13]. Among these ncRNAs, lncRNAs are a class of transcripts with lengths greater than 200 nucleotides. The lncRNAs are emerging as a significant regulator responsible for various biological processes, such as cell growth, cell migration, cell invasion, and metabolic rewiring [14–16]. These abnormally expressed lncRNAs have been implicated as potential alternative biomarkers or therapeutic targets for NSCLC [17, 18]. Although a growing number of lncRNAs have been annotated, the specific roles and molecular mechanisms of lncRNAs in regulating aerobic glycolysis of NSCLC remain poorly understood [19].

In the current study, RNA sequencing analysis was performed between NSCLC tumor tissues with high ^18^F-FDG uptake and their corresponding noncancerous tissues. An upregulated lncRNA—LINC01123—was found to exert oncogenetic function in promoting proliferation as well as glycolysis. Moreover, LINC01123 was directly transcribed by c-Myc and inversely increased c-Myc expression level. This study indicated the existence of a positive feedback loop between LINC01123 and c-Myc, suggesting a novel mechanism in interpreting metabolic reprogramming and malignant progression of NSCLC.

## Materials and methods

### Clinical samples and RNA sequencing assay

Two independent cohorts were enrolled. Cohort 1: Fresh NSCLC tumor tissues and adjacent tissues (5 cm from the tumor edge) were obtained from three patients in November 2017 at Shanghai Chest Hospital. Cohort 2: Specimens from 92 patients who underwent surgery between January 2008 and July 2013 were acquired from the surgical specimen archives of Renji Hospital, School of Medicine, Shanghai Jiaotong University, and patients’ follow-up visit continue to June 2018. The data was censored at the last follow-up visit or at the time of the patient’s death without relapse. No recruited patients received any preoperative treatment. All patients were staged based on the criteria of the 8th Edition of the Lung Cancer Staging Manual [5]. This research was approved by the institutional clinical research ethics committee of Shanghai Chest Hospital and Renji Hospital, School of Medicine, Shanghai Jiaotong University. Written informed consent was obtained from each patient, and the study was conducted in accordance with the International Ethical Guidelines for Biomedical Research Involving Human Subjects (CIOMS).

Next-generation RNA sequencing assay was performed to detect the mRNA and ncRNA expression profiles at KangChen Bio-tech (Shanghai, China) using Illumina HiSeq 4000 (Illumina, San Diego, CA, USA). Solexa pipeline version 1.8 was used to align the reads to the genome, generate raw counts corresponding to each known gene (a total of 17,242 genes), and calculate the RPKM (reads per kilobase per million) values. The differential expression lncRNAs and mRNAs were identified through fold change/*p* value/FDR filtering (fold change ≥ 1.5, *P* value < 0.05, and FDR < 0.05).

### Total RNA extraction and qRT-PCR

Total RNA was isolated by TRIzol Kit (Omega, Norcross, GA, USA), and the quantity of total RNA was measured by using a NanoDrop equipment (Thermo Fisher Scientific, Waltham, MA, USA). Complementary DNA (cDNA) was synthesized using the cDNA Synthesis kit (Takara, Otsu, Japan). Real-time PCR was performed using SYBR Green PCR Master Mix (Takara) in a StepOnePlus RT-PCR system (Thermo Fisher Scientific). All target genes were normalized to the endogenous reference gene β-actin by using an optimized comparative Ct (2^ΔΔCt^) value method. To measure the level of miR-199a-5p, miRNA extraction kit (Vazyme, Nanjing, China) and a stem-loop miRNA Synthesis Kit (Vazyme) were used according to the manufacturer’s instructions. U6 snRNA was used as the endogenous control. The sequences of primers used in this study are listed in Additional file 7: Table S1.

### Subcellular RNA fractionation

Subcellular fractionation was performed with a PARIS™ Kit (Ambion, Austin, TX) according to the manufacturer’s instructions. The nuclear and cytoplasmic RNA was further analyzed by qPCR. β-Actin and U6 were used as cytoplasmic and nuclear controls, respectively.

### Fluorescence in situ hybridization

Fluorescence in situ hybridization (FISH) analysis was performed according to a previously described method [20]. Briefly, specific target probes were made for LINC01123. The hybridization was performed using RNAscope Fluorescent Reagent Kit (Advanced Cell Diagnostics, Hayward, CA, USA) according to the manufacturer’s instructions. Staining score was assessed by two independent reviewers as 0 = no staining, 1 = weak staining, 2 = moderate staining, and 3 = strong staining. Tumor cells in five fields were selected randomly and scored based on the percentages of positively stained cells (1 = 0–25%, 2 = 26–50%, 3 = 51–75%, 4 = 76–100%). The final scores were computed by multiplying the intensity score and the percentage score of positive cells. According to the score, samples were divided into two groups, the negative and low expression group (score 0–6) and high expression group (score 7–12).

### Cell lines and culture condition

Five NSCLC cell lines, A549, H1299, H1650, H1975, and PC9, human normal lung epithelial cell line HBE, and human diploid fibroblast IMR-90 cells were purchased from ATCC (Manassas, VA, USA) and cultured in Dulbecco’s modified Eagle’s medium (DMEM), supplemented with 10% fetal bovine serum, 100 U/mL penicillin, and 100 μg/mL streptomycin (GIBCO, Grand Island, NY, USA) at 37 °C with 5% CO_2_. All cells were tested for mycoplasma contamination and had no mycoplasma contamination.

### Plasmids, oligonucleotides, siRNA, and transfection

LINC01123 pcDNA3.1 vector (1123-OE), c-Myc pcDNA3.1 vector (Myc-OE), and empty vector (vector) were subcloned into the expression vector pcDNA3.1 (Invitrogen, USA). MiR-199a-5p mimic, negative control oligonucleotides (mimics NC), miR-199a-5p inhibitor, negative control oligonucleotide (inhibitor NC), small interfering RNA of LINC01123 or c-Myc (si-1123, si-Myc), and scramble siRNA (si-NC) were purchased from GenePharma (Shanghai, China). The cells were seeded into six-well plates and cultured overnight until 70–80% confluence. Transfection was performed using Lipofectamine 2000 (Invitrogen, Carlsbad, CA, USA) according to the manufacturer’s instruction. The transfection efficiency was determined using qRT-PCR and Western blot. Sh-1123 and sh-NC lentivirus were purchased from GenePharma (Shanghai, China) and constructed into A549 cell lines for further in vivo experiments. The transfection efficiency was evaluated by fluorescence microscopy by calculating the percentage of fluorescein-labeled cells.

### Cell proliferation assay and colony formation assay

The relative cell viability at 24, 48, and 72 h after transfection was monitored using the Cell Counting Kit-8 (CCK-8, Bimake, Shanghai, China) according to the manufacturer’s protocol. Briefly, 5 × 10^3^ cells were cultured in a 96-well plate at 37 °C. After 10 μL CCK-8 solution was added to each well, plates were incubated at 37 °C for 1 h. After that, the optical density at 450 nm (OD450) was measured for each sample.

As for the colony formation assay, a total of 500 cells were seeded in 6-well plates and cultured in a humidified atmosphere containing 5% CO_2_ at 37 °C for 2 weeks. Cell colonies were washed with PBS, fixed with 4% paraformaldehyde, and stained with 0.1% crystal violet (1 mg/mL) for 20 min. All the experiments were repeated in triplicate, and the mean was calculated.

### Ethynyldeoxyuridine analysis

Ethynyldeoxyuridine (EdU) detection kit (RiboBio, Guangzhou, China) was used to assess cell proliferation according to the manufacturer’s instruction. Cells were cultured in 96-well plates at 5 × 10^3^ cells/well. Ten microliters of EdU labeling media was added to the 96-well plates and then incubated at 37 °C under 5% CO_2_ for 2 h. After treatment with 4% paraformaldehyde and 0.5% Triton X-100, the cells were stained with the anti-EdU working solution and Hoechst 33342. Subsequently, the cells were visualized using a fluorescence microscope (Olympus, Tokyo, Japan). The EdU incorporation rate was calculated as the ratio of the number of EdU-positive cells (green cells) to the total number of Hoechst 33342-positive cells (blue cells).

### Glucose uptake, lactate production, LDH enzyme activities, intracellular ATP, and ROS level

As an analog of glucose, ^18^F-FDG uptake assay could reflect the intracellular glucose uptake level of cells. The cells were seeded into 12-well plates at 1 × 10^5^ cells/well and cultured overnight. After the culture medium was removed and washed twice with PBS, cells were incubated in 1 mL of glucose-free DMEM containing 74–148 kBq/mL (2–4 μCi/mL) ^18^F-FDG for 1 h at 37 °C. Then, wash the cells twice with PBS and add 1 mL 0.5 M NaOH per well to produce cell lysates. A well γ-counter was used to detect the radioactivity of lysates. The intracellular ^18^F-FDG uptake was radioactive readouts normalized to corresponding cell numbers. Three independent experiments were performed during our study.

For lactate production measurements and LDH enzyme activity, cell supernatant was collected to measure lactate concentration (Nanjing Jiancheng Bioengineering Institute, Nanjing, China), while the cell pellets to be lysed and measured ATP level (Nanjing Jiancheng) according to the manufacturer’s instructions. ROS was measured by fluorescent 2′, 7′-dichlorofluorescin diacetate (DCF-DA) as described by manufacturer’s protocol of a commercial kit (Nanjing Jiancheng).

### Extracellular acidification rate (ECAR) and oxygen consumption rate (OCR) assays

The extracellular acidification rate (ECAR) and oxygen consumption rate (OCR) were measured using the Seahorse XF 24 Extracellular Flux Analyzer (Seahorse Bioscience), according to a previously described method [20]. ECAR and OCR were measured using Seahorse XF Glycolysis Stress Test kit and Seahorse XF Cell Mito Stress Test kit (Agilent Technologies), respectively.

### Xenograft mouse model and micro-PET/CT

Tumorigenicity assays in nude mice were performed. Our study was approved by Renji Institutional Animal Care and Use Committee (Shanghai, China). The procedures were performed according to the guidelines and regulations of Renji Hospital, School of Medicine, Shanghai Jiaotong University (Shanghai, China). This study was carried out in accordance with the recommendations that cover all scientific procedures involving the use of live animal. Briefly, 4-week male BALB/c nude mice were purchased from Renji Hospital Experimental Animal Center (Shanghai, China). The mice were inoculated subcutaneously with 1 × 10^7^ cells in the right flank with sh-1123, while sh-NC in the left flank.

After 3 weeks, a micro-PET/CT scanner (Super Nova® PET/CT, PINGSENG Healthcare Inc., Shanghai, China) was used to measure ^18^F-FDG uptakes in the mice. PET/CT scanner is ~ 0.6 mm, and the resolution of the CT is 0.2 mm. ^18^F-FDG (0.2 mL, 7.4 MBq) was injected into the tail vein of tumor-bearing mice. After 30 min, the animals were anesthetized with 2% isoflurane and immobilized during 20 min PET scan acquisition. PET images were reconstructed with the ordered-subsets expectation maximization (OSEM) algorithm using 16 subsets and 4 iterations. An irregular region of interest, which covered the entire tumor, was drawn on the CT and then copied to the co-registered PET using Avatar 1.2 software (Pingseng, Shanghai, China). ^18^F-FDG uptake by tumors was assessed by the maximum standard uptake value (SUVmax). After PET/CT scan, mice were sacrificed and tumors were excised and weighed.

### Immunohistochemistry

Immunohistochemistry (IHC) was performed and measured as reported previously [20].

### Western blot analysis

Cells were lysed using radioimmunoprecipitation assay (RIPA) lysis solution (50 mM Tris-HCl, pH 7.4, 150 mM NaCl, 1% NP-40, and complete protease inhibitor cocktail) for 30 min on ice, then centrifuged at 15,000×*g* for 30 min at 4 °C. Cell extracts were boiled for 5 min at 100 °C, and the protein samples were analyzed by Western blot. Western blot analysis was cultured as previously described [21]. c-Myc polyclonal antibody (Proteintech, Chicago, USA), HK2 and LDHA polyclonal antibody (Proteintech), and anti-β-actin monoclonal antibody (Proteintech) were used according to the manufacturer’s protocol. Immunoreactive bands were visualized using ECL Western blot kit (Amersham Biosciences, Buckinghamshire, UK).

### Immunofluorescence (IF)

Cells were cultured in six-well plates on glass coverslips, fixed for 20 min with 4% formaldehyde, and permeabilized with 0.25% Triton X-100; the cells were treated with blocking buffer for 30 min and incubated overnight at 4 °C with the c-Myc polyclonal antibody (1:500, Proteintech), followed by incubation with the secondary antibody at room temperature for 1 h. Cell nuclei were counterstained with DAPI. Confocal laser scanning microscope (Olympus BX61) was used to observe the image.

### Luciferase reporter assay

To determine the effect of c-Myc on LINC01123 promoter, pcDNA-c-Myc, pcDNA-vector, si-Myc, or si-NC was individually co-transfected into 293 T cells together with the pGL3-based construct containing LINC01123 WT or MUT promoter sequences plus Renilla luciferase reporter plasmid. Twenty-four hours after transfection, firefly and Renilla luciferase activity were measured by a Dual-Luciferase Reporter Assay System (Promega, Madison, WI, USA). The ratio of firefly luciferase to Renilla activity was calculated for each sample. To evaluate the effect of miR-199a-5p on LINC01123 or c-Myc 3′UTR, 293 T cells were co-transfected with the pmirGLO-LINC01123-WT, pmirGLO-LINC01123-MUT, pmirGLO-c-Myc-3′UTR-WT, or pmirGLO-c-Myc-3′UTR-MUT reporter plasmids individually together with mimics NC, miR-199a-5p mimics, inhibitor NC, or miR-199a-5p inhibitor, respectively. To confirm the competing binding of miR-199a-5p between LINC01123 and c-Myc 3′UTR, pcDNA-LINC01123 or pcDNA-vector was co-transfected with miR-199a-5p mimics and pmirGLO-c-Myc-3′UTR-WT or pmirGLO-c-Myc-3′UTR-MUT, respectively. Si-1123 or si-NC was co-transfected with miR-199a-5p inhibitor and pmirGLO-c-Myc-3′UTR-WT or pmirGLO-c-Myc-3′UTR-MUT, respectively. Twenty-four hours after transfection, firefly and Renilla luciferase activity were measured by a Dual-Luciferase Reporter Assay System (Promega). Experiments were performed in triplicate, and the data are represented as mean SD.

### Chromatin immunoprecipitation assay

Chromatin immunoprecipitation (ChIP) was performed with a ChIP assay kit (Beyotime, Haimen, Jiangsu, China) according to the manufacturer’s protocol. Briefly, A549 and H1299 cells were cross-linked with 1% formaldehyde for 10 min and sonicated to shear DNA to lengths between 200 and 1000 base pairs. Cell lysates were precleared with protein A/G beads before they were incubated at 4 °C overnight with protein A/G beads coated with the anti-c-Myc antibody (2 μg, Proteintech). Anti-rabbit immunoglobulin G (IgG) was also used as a negative control. After extensive washing, the bead-bound immunocomplexes were eluted using elution buffer. To reverse histone-DNA crosslinks, samples were added with 5 M NaCl and heated at 65 °C for 4 h, then treated with proteinase K and further incubated at 45 °C for 1 h. The bound DNA fragments were purified by DNA Extraction Kit (GeneMark, Shanghai, China) and subjected to real-time PCR using the specific primers.

### RNA immunoprecipitation assay

RNA immunoprecipitation (RIP) was performed using a Magna RNA-binding protein immunoprecipitation kit (Millipore, Bedford, MA, USA) according to the manufacturer’s instructions. Briefly, cells were collected and lysed in complete RIPA buffer containing a protease inhibitor cocktail and RNase inhibitor. Next, the cell lysates were incubated with RIP buffer containing magnetic bead conjugated with human anti-Ago2 antibody (Proteintech) or control normal mouse IgG. The samples were digested with proteinase K to isolate the immunoprecipitated RNA. The purified RNA was finally subjected to real-time PCR to demonstrate the presence of the binding targets.

### Statistical procedures

Statistical analysis was performed using SPSS 20.0 software (SPSS, Inc., Chicago, IL) and GraphPad Prism 7.0 (GraphPad Software, Inc., USA). The results are presented as mean ± SD. Comparison between two groups was assessed using Student’s *t* test (two-tailed, with *P* < 0.05 considered significant). The chi-square test, one-way analysis of variance, and Pearson’s correlation were also performed. The survival curves were calculated using the Kaplan-Meier method, and the differences were assessed by a log-rank test. Cox multivariate regression analysis was used to determine the independent factors that influenced survival and recurrence. All results were reproduced across triplicate experiments. One asterisk, two asterisks, and three asterisks indicate *P* < 0.05, *P* < 0.01, and *P* < 0.001, respectively.

## Results

### LncRNA expression profiles in NSCLC

**Fig. 1:**
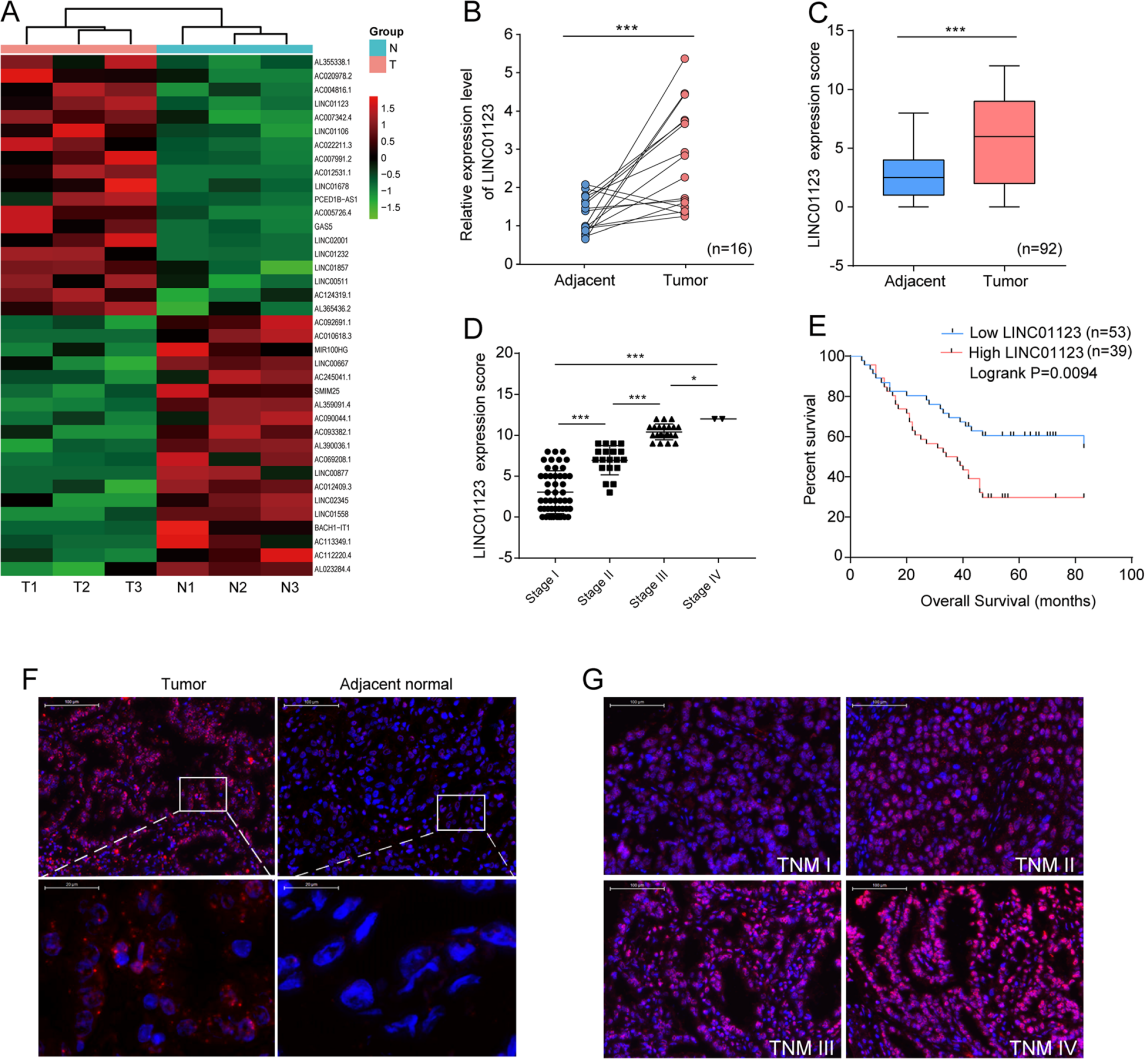
LINC01123 is upregulated in NSCLC tissues and predicts poor prognosis. **a** The heat map reflects the top 20 upregulated and downregulated lncRNAs in RNA-seq analysis of 3 paired NSCLC tumor and adjacent tissues. **b** Expression of LINC01123 in 16 paired NSCLC tissues was analyzed by qRT-PCR. **c** The relative LINC01123 expression score analyzed by FISH in NSCLC tissues and adjacent normal tissues (*n* = 92). **d** Relative LINC01123 expression levels in different TNM stages. **e** Kaplan-Meier analysis showed that elevated expression of LINC01123 was associated with overall survival (OS) in NSCLC patients. **f**, **g** Representative FISH images of LINC01123 expression in NSCLC tumor tissues and in different TNM stages (blue, DAPI; red, positive staining). Data shown are mean ± SD (*n* = 3). (*P < 0.05, **P < 0.01, ***P < 0.001)

Total RNA of three high ^18^F-FDG uptake tumor tissues and their corresponding normal lung tissues extracted from NSCLC patients (Additional file 1: Figure S1A-C) were analyzed by RNA-seq. After screening (fold change ≥ 1.5, *P* value< 0.05, and FDR < 0.05), the expression profiling data suggested 364 differentially expressed genes, including 222 upregulated and 142 downregulated genes in total (Additional file 2: Figure S2A). The hierarchical clustering of top 20 upregulated and downregulated lncRNAs is listed in Fig. 1a. KEGG pathway enrichment analysis for the top 10 significantly upregulated lncRNA/mRNAs demonstrated that they were associated with 10 pathways. Metabolic pathway was one of the most upregulated pathways (Additional file 2: Figure S2B-C). GSEA analysis showed that the aberrant lncRNA/mRNAs mainly took part in the glycolysis and gluconeogenesis biological processes (Additional file 2: Figure S2D). The top 10 upregulated and downregulated lncRNA/mRNAs are listed in Additional file 8: Table S2, respectively.

To verify the reliability of RNA-Seq results, 16 paired NSCLC tissues and the corresponding adjacent lung tissues were collected to validate 7 randomly selected upregulated lncRNAs by qRT-PCR assay. In agreement with RNA-Seq results, LINC01123 (accession: NR_046110, a 2519 bp transcript with 4 exons and localizes in human chromosome 2q13) was markedly increased in tumor tissues compared with adjacent non-tumor tissues (Fig. 1b, Additional file 3: Figure S3A), and the expression of other 6 lncRNAs were consistent with those observed in RNA-Seq (Additional file 2: Figure S2E-J).Additionally, the coding potential of LINC01123 was predicted using several prediction software and the NCBI ORF finder (https://www.ncbi.nlm.nih.gov/orffinder/); the results showed that LINC01123 could not code any protein, indicating it a non-coding RNA (Additional file 3: Figure S3B-D).

### Expression of LINC01123 in NSCLC tissues and its clinical significance

To assess the clinical significance of LINC01123 expression, ISH was performed on 92 pairs of NSCLC tissues and the adjacent non-tumor tissues. The results showed that LINC01123 expression was markedly higher in tumor tissues and increased with advanced TNM stage (Fig. 1c, d, f, g). The 92 paired samples were then stratified into high expression group (score 7–12) and low expression group (score 0–6) based on LINC01123 expression score. As shown in Table 1, LINC01123 expression was significantly associated with tumor stage (*P* = 0.002), lymph node metastasis (*P* = 0.002), and TNM stage (*P* = 0.001). However, no correlation was observed between LINC01123 and age, gender, distant metastasis, histological grade, or tumor location. Kaplan-Meier analysis revealed that the expression level of LINC01123 was significantly associated with overall survival (OS) (*P* = 0.0094, Fig. 1e). In addition, univariate analysis revealed that tumor grade, lymph node metastasis, and TNM stage were also associated with overall survival. Cox proportional hazard regression analysis further demonstrated that LINC01123 expression (HR = 2.029; 95% CI 1.140–3.613; *P* = 0.016) and TNM stage (HR = 2.576; 95% CI 1.252–5.300; *P* = 0.010) were independent prognostic indicators for NSCLC patients (Table 2).

**Table 1:** Correlation of the expression of LINC01123 in NSCLC with clinicopathologic features

Characteristics		LINC01123		*P* value
		Low and negative (*n*)	High (*n*)	
Age (years)				
< 55	32 (34.8%)	17	15	0.827
≥ 55	60 (65.2%)	29	31	
Gender				
Male	51 (55.4%)	26	25	0.500
Female	41 (44.6%)	20	21	
TNM stage				
I	49 (53.3%)	32	17	0.001**
II	19 (20.7)	10	9	
III	22 (23.9%)	4	18	
IV	2 (2.2%)	0	2	
T stage				
T1	37 (40.2%)	21	16	0.002**
T2	37 (40.2%)	21	16	
T3	11 (12.0%)	3	8	
T4	7 (7.6%)	1	6	
Lymph node metastasis				
N0	55 (59.8%)	33	22	0.002**
N1	22 (23.9%)	9	13	
N2	11 (12.0%)	4	7	
N3	4 (4.3%)	0	4	
Distant metastasis				
M0	90 (97.8%)	46	44	0.495
M1	2 (2.2%)	0	2	
Mortality				
Survive	41 (44.6%)	27	14	0.011*
Die	51 (55.4%)	19	32	
Histological grade				
I	2 (2.2%)	2	0	0.467
II	57 (62.0%)	25	32	
III	33 (35.9%)	19	14	
Tumor location				
Left	43 (46.7%)	24	19	0.454
Right	49 (53.3%)	22	27	

*P < 0.05; **P < 0.01

**Table 2:** Univariate and multivariate Cox regression analysis for clinicopathological features associated with prognosis of 92 NSCLC patients

Variables	Univariate analysis		Multivariate analysis	
	HR (95% CI)	*P*	HR (95% CI)	*P*
Age (≥ 55 vs. < 55)	1.294 (0.715–2.339)	0.394	1.243 (0.677–2.282)	0.483
Gender (male vs. female)	1.445 (0.823–2.537)	0.200	1.562 (0.886–2.754)	0.123
TNM stage (I–II vs. III–IV)	3.045 (1.720–5.391)	0.001**	2.576 (1.252–5.300)	0.010*
T stage (T1–2 vs. T3–4)	2.300 (1.233–4.290)	0.009**	1.575 (0.768–3.230)	0.215
Lymph node metastasis (N0 vs. N1–3)	1.823 (1.051–3.163)	0.033*	1.790 (0.919–3.486)	0.087
Distant metastasis (M0 vs. M1)	0.393 (0.094–1.641)	0.200	0.540 (0.127–2.287)	0.403
Histologic grade (I–II vs. III)	1.373 (0.782–2.411)	0.269	1.375 (0.783–2.415)	0.268
Tumor location (left vs. right)	0.936 (0.530–1.654)	0.820	0.913 (0.516–1.615)	0.754
LINC01123 (low and negative vs. high)	2.085 (1.179–3.689)	0.012*	2.029 (1.140–3.613)	0.016*

*P < 0.05; **P < 0.01

Furthermore, expression levels of LINC01123 were analyzed in the GEO dataset (GSE19804) from R2: Genomics Analysis and Visualization Platform (http://r2.amc.nl) and TCGA dataset from StarBase V3.0 [22]. Both datasets showed that LINC01123 expression was significantly upregulated in NSCLC tumor tissues compared to normal tissues (Additional file 4: Figure S4A-B). TCGA data from GEPIA Platform (http://gepia.cancer-pku.cn/) also demonstrated that high LINC01123 expression indicated poor survival of NSCLC patients (*P* < 0.05, Additional file 4: Figure S4C-D). Collectively, these results suggested that LINC01123 might be a potential biomarker in NSCLC.

### LINC01123 facilitates proliferation and metabolic rewiring of NSCLC cells in vitro

**Fig. 2:**
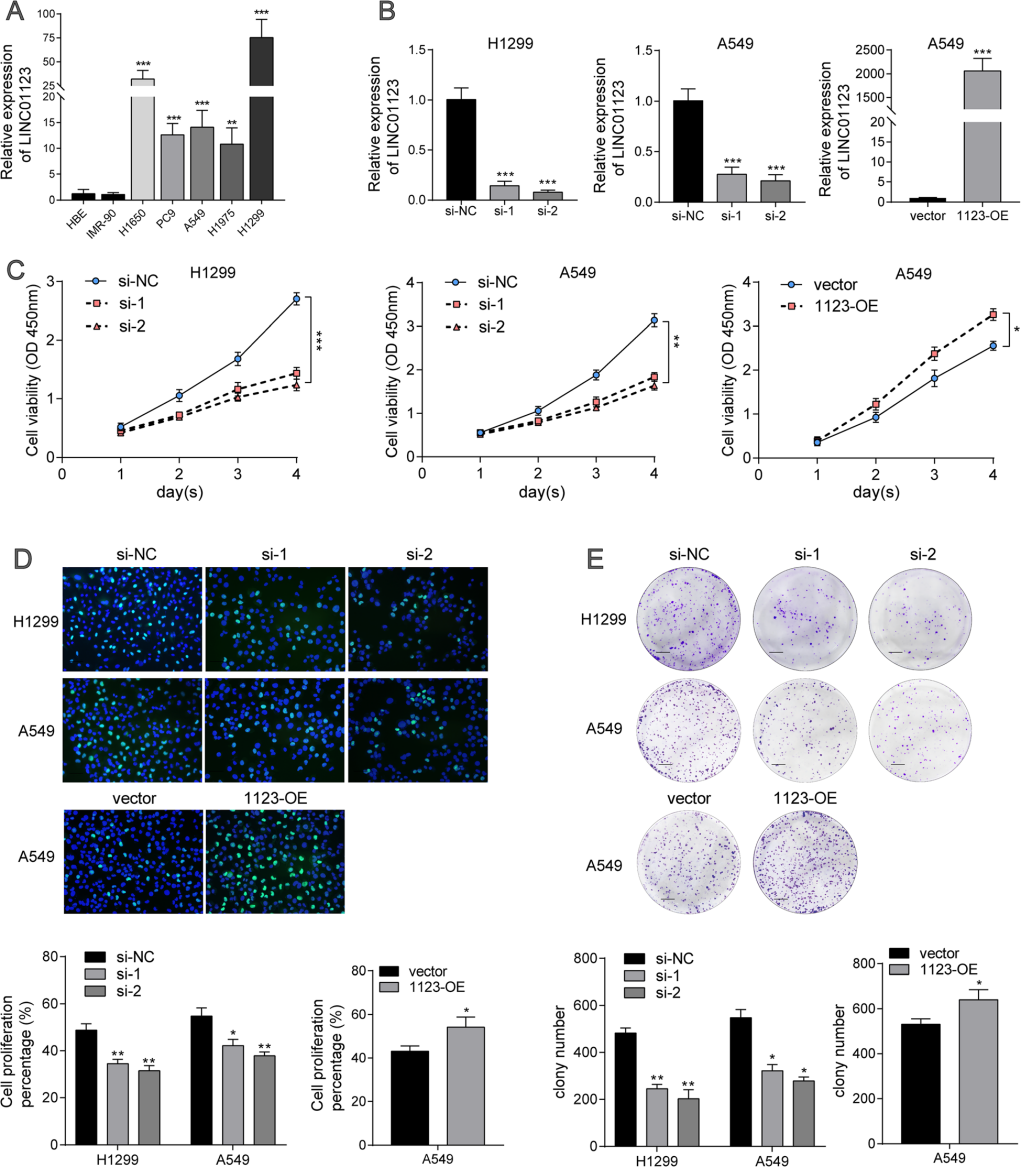
LINC01123 facilitates NSCLC cell proliferation in vitro. **a** Expression of LINC01123 in five NSCLC cell lines (A549, H1299, H1650, H1975, PC9) and two normal lung cell line (HBE and IMR-90) was analyzed by qRT-PCR. **b** The expression of LINC01123 was knocked down by two different siRNAs in both H1299 and A549 cells. Relative expression levels of LINC01123 were significantly upregulated in A549 cell after transfection with pcDNA-1123. **c**–**e** Cell proliferation was analyzed by CCK-8 assay, colony formation, and immunofluorescence analysis with Edu. Knockdown of LINC01123 expression significantly inhibited cell proliferation in H1299 and A549 cells, while ectopic induced of LINC01123 promoted cell proliferation in A549 cells. Scale bar = 100 μm (colony) or 20 μm (Edu). Data shown are mean ± SD (*n* = 3). (*P < 0.05, **P < 0.01, ***P < 0.001)

To elucidate the function of LINC01123 on cell biological behavior, we first explored the expression level of LINC01123 in five NSCLC cell lines compared with normal lung cells IMR-90 and HBE (Fig. 2a). Then, we transfected the H1299 and A549 cells with two different siRNAs and each siRNA could effectively knock down LINC01123 expression. Si-2 showed the strongest suppression of LINC01123 (> 90% inhibitory rate) and therefore was used throughout this study. The whole length of LINC01123-pcDNA overexpression vector was constructed and transfected into A549 cell. Following, qRT-PCR verified that the expression of LINC01123 could increase over 2000 fold (Fig. 2b). To investigate the biological roles of LINC01123 in NSCLC progression, the cell proliferation was analyzed using CCK-8 assay, clone formation, and EdU staining. All these results showed that A549 cell proliferative capability was increased following LINC01123 overexpression, while knockdown of LINC01123 remarkably attenuated the proliferative effects in H1299 and A549 cells (Fig. 2c–e). It can be concluded that LINC01123 is effective in facilitating NSCLC cell growth.

**Fig. 3:**
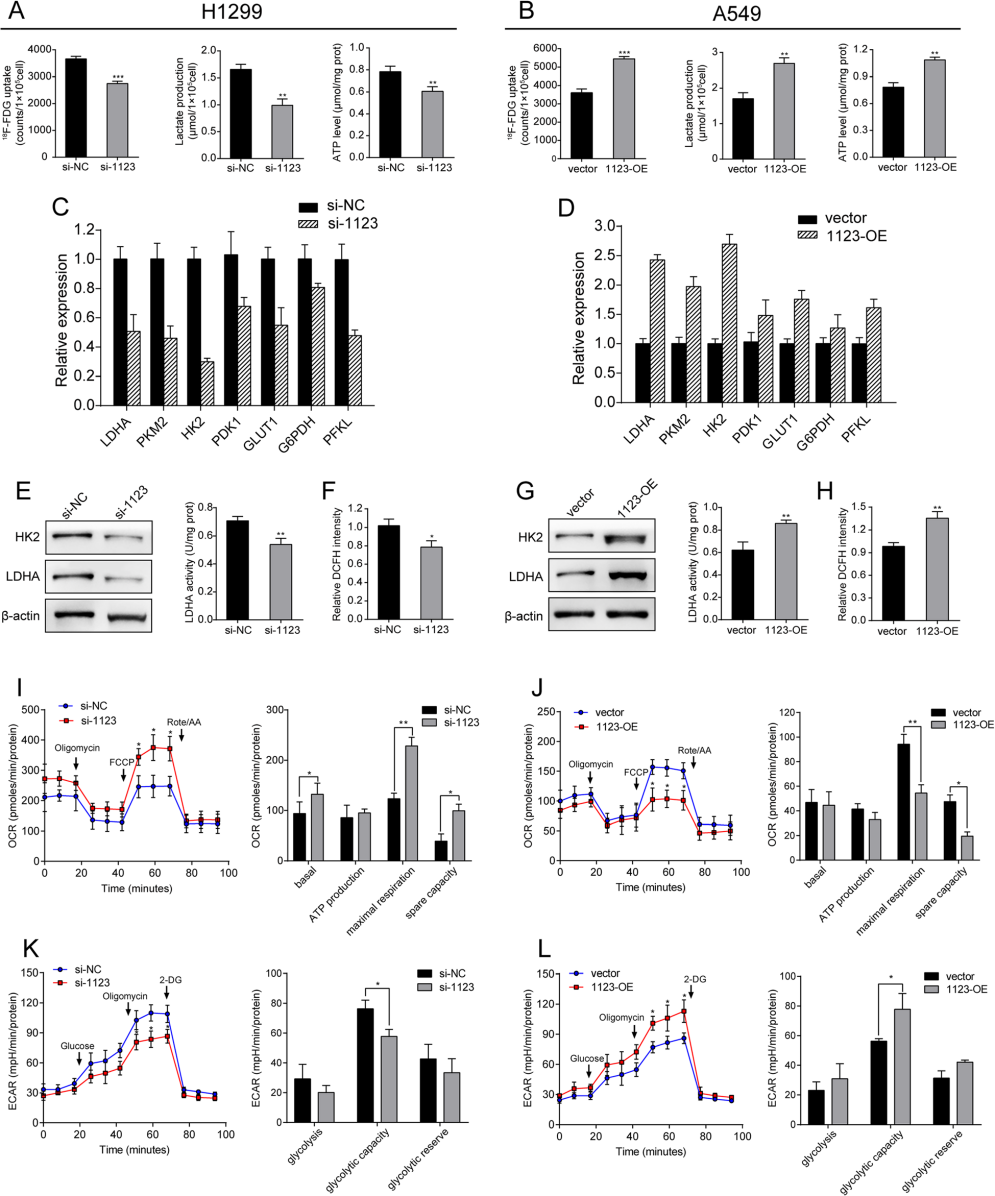
LINC01123 enhances NSCLC cells metabolic rewiring in vitro. **a**, **b** ^18^F-FDG uptake, lactate release, and ATP production level were determined in H1299 and A549 cells. **c**, **d** Relative expression levels of metabolic genes in H1299 cells after being transfected with si-1123 and in A549 cells after transfection with pcDNA-1123. **e**–**h** HK2 and LDHA protein expression, LDH enzyme activity, and ROS accumulation were examined in H1299 and in A549 cells. **i**–**l** The quantification of OCR and ECAR measured by Seahorse XF assays in H1299 and A549 cells with LINC01123 knockdown (**i**, **k**) or overexpression (**j**, **l**).The OCR curves were treated with oligomycin, FCCP, and rotenone/antimycin A, while the ECAR curves were treated with glucose, oligomycin, and 2-DG. Data shown are mean ± SD (*n* = 3). (*P < 0.05, **P < 0.01, ***P < 0.001)

It is well documented that aerobic glycolysis is essential for cancer cell proliferation and growth. Malignant progression of cancer could activate multiple carcinogenic signals contributing to metabolic reprogramming. In our study, LINC01123 was an upregulated gene associated with high ^18^F-FDG uptake on PET/CT image of NSCLC patients. Consistent with this, we hypothesized whether LINC01123 had functions in regulating the processing of glycolysis. The results showed that LINC01123 induction led to an increase in ^18^F-FDG uptake, lactate, and ATP production. In contrast, knockdown of LINC01123 led to opposite results (Fig. 3a, b). To further confirm the effect of LINC01123 on glycolysis, we examined mRNA expression level of several glucose metabolic enzymes and protein expression level of key glycolytic enzymes HK2 and LDHA was also increased, as well as enzyme activity of LDHA (Fig. 3c–e and g). Since enhanced glycolysis might facilitate ROS homeostasis, ROS level was examined and results confirmed that overexpression of LINC01123 induced ROS accumulation, whereas LINC01123 knockdown abrogated this effect (Fig. 3f, h). In addition, knockdown of LINC01123 also displayed decreased extracellular acidification rate (ECAR), which reflects overall glycolytic flux, and increased oxygen consumption rate (OCR), an indicator of mitochondrial oxidative respiration. In contrast, overexpression of LINC01123 reversed these effects (Fig. 3i–l). All the above findings uncovered a novel function of LINC01123 in regulating metabolic reprogramming of NSCLC cells.

### LINC01123 promotes tumor growth and aerobic glycolysis in vivo

**Fig. 4:**
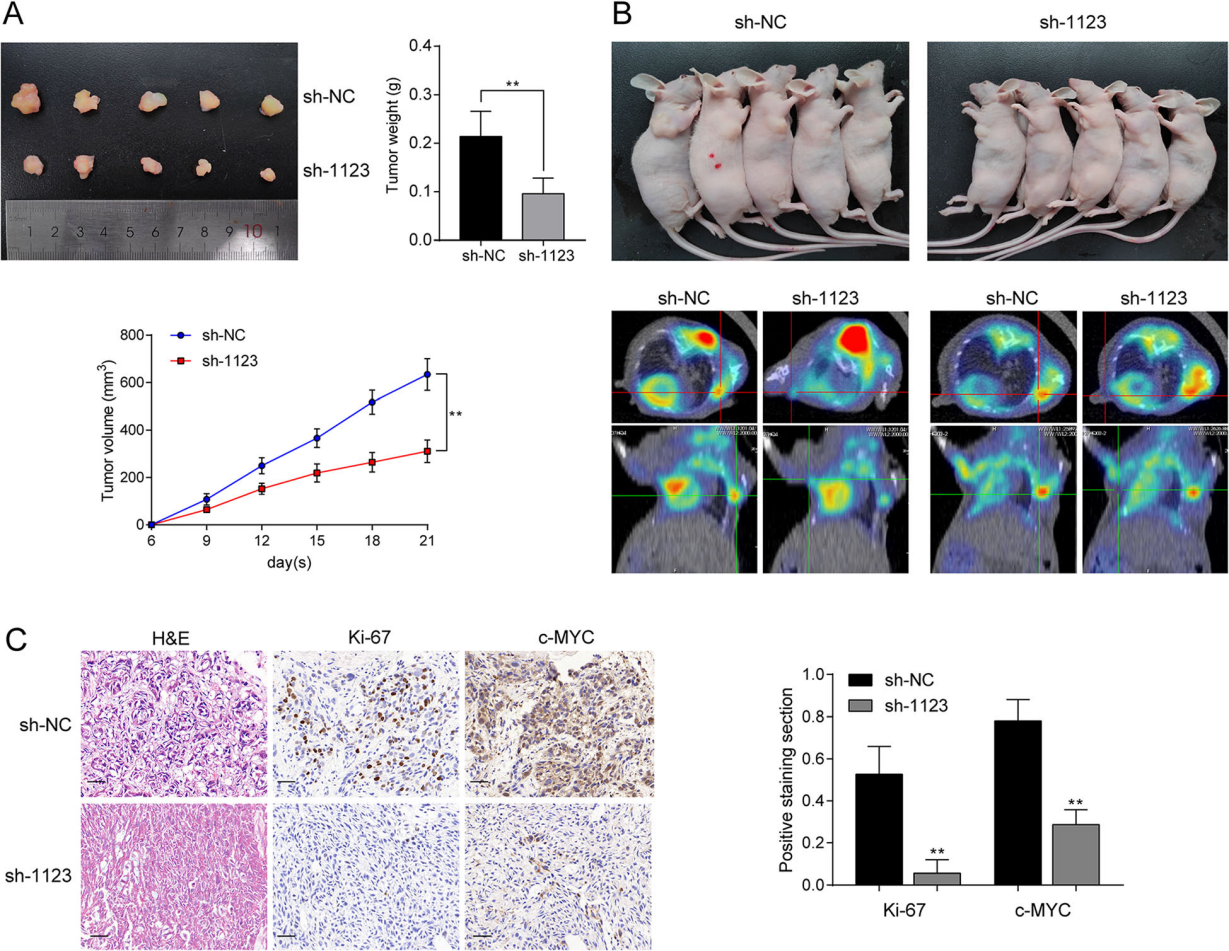
LINC01123 promotes tumor growth and aerobic glycolysis in vivo. **a** A549 cell treated with sh-NC lentivirus were inoculated subcutaneously into the left flanks and treated with sh-1123 lentivirus into the right flanks of nude mice (*n* = 5). Tumor growth curves showed that sh-1123 group suppressed tumor growth compared with sh-NC group. **b** ^18^F-FDG micro-PET/CT images of living nude mice were conducted 3 weeks after injection. Images showed obvious FDG uptake in sh-NC xenografts, while mild FDG uptake in sh-1123 xenografts. **c** The xenografts were subjected to H&E and IHC staining with Ki-67 and c-Myc. Scale bar = 50 μm (× 400). Data shown are mean ± SD (*n* = 3). (*P < 0.05, **P < 0.01, ***P < 0.001)

The effect of LINC01123 on tumor growth in vivo was examined using mouse subcutaneous xenograft models. Tumor growth was slower in sh-1123 group with smaller tumor volumes and lower tumor weights than sh-NC group (Fig. 4a). Additionally, the glucose uptake ability of the xenograft was detected by ^18^F-FDG micro-PET/CT imaging. Results showed that xenografts derived from sh-NC group had relatively strong accumulation of ^18^F-FDG (mean SUVmax 4.75), while ^18^F-FDG uptake in sh-1123 group was much lower (mean SUVmax 1.08; Fig. 4b). To further validate the ability of LINC01123 in mediating tumor growth, the xenograft tissues were stained with ki-67 and c-Myc antibody for IHC analysis (Fig. 4c). Collectively, these in vivo experiment results were consistent with in vitro data that LINC01123 knockdown decreased tumor growth and glycolysis ability of NSCLC cells.

### LINC01123 is a direct transcriptional target of c-Myc

**Fig. 5:**
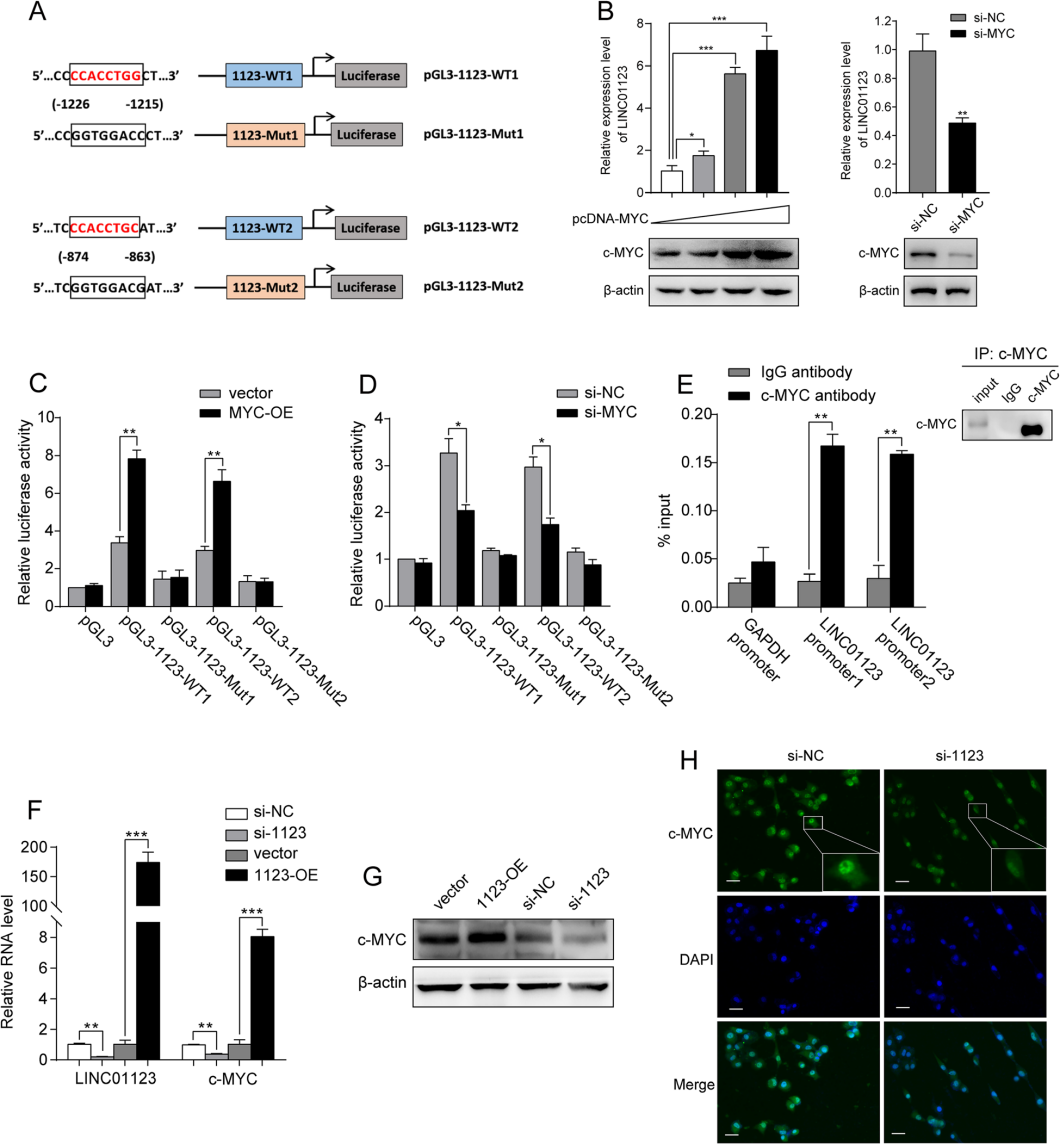
LINC01123 is a direct transcriptional target of c-Myc and inversely enhances c-Myc level. **a** Schematic illustration of consensus c-Myc binding sites in LINC01123 gene promoter. The indicated pGL3-based luciferase reporters were constructed containing either of the two putative c-Myc binding sites or their corresponding mutant binding sites, which were shown in the open boxes. **b** A549 cell was transfected with 0.5 μg, 1 μg, or 2 μg pcDNA-c-Myc. H1299 cell was transfected with si-NC or si-Myc. Forty-eight hours after transfection, cell lysates and total RNA were subjected to Western blot and qRT-PCR analyses, respectively. **c**, **d** Two hundred ninety-three T cell was co-transfected with either pcDNA c-Myc or si-Myc plus the indicated reporter constructs and Renilla luciferase plasmid. Twenty-four hours after transfection, reporter activity was measured and plotted after normalizing with respect to Renilla luciferase activity. **e** Lysates from A549 cell were subjected to ChIP assay. ChIP products were amplified by qPCR with the indicated pairs of primers. **f**–**h** QRT-PCR, Western blot, and immunofluorescence showed that c-Myc mRNA and protein level were positively regulated by LINC01123 in A549 and H1299 cells. Data shown are mean ± SD (*n* = 3). (*P < 0.05, **P < 0.01, ***P < 0.001)

We next explored which factors induced high LINC01123 expression in NSCLC. The genomic sequence upstream regions (~ 2 kb upstream) of the gene coding for LINC01123 were inspected using the promoter sequence analysis tools (UCSC and JASPAR). Two putative c-Myc binding sites (CCACCTG/C) were found within the promoter region of the LINC01123 gene (Fig. 5a). The qRT-PCR analysis showed that levels of LINC01123 was greatly increased with c-Myc overexpression and presented a dose-dependent tendency, while knockdown of c-Myc decreased LINC01123 expression (Fig. 5b). Then, whether c-Myc directly interact with the two promoter binding sites was then investigated. DNA fragments containing wild-type or mutant binding sequence were inserted into the promoter region of a firefly luciferase reporter plasmid. As was expected, luciferase activity from the reporter containing the two individual wild-type binding sites was induced by ectopic expression of c-Myc and decreased by knockdown of c-Myc. Yet, the reporter containing mutant sites showed no response to c-Myc induction or silencing (Fig. 5c, d). The subsequent chromatin immunoprecipitation followed by qPCR (ChIP–qPCR) assays supported the notion that c-Myc directly binds to the chromatin fragments of the two predicted promoter regions of LINC01123 gene and further regulated LINC01123 transcription (Fig. 5e).

### LINC01123 enhances c-Myc expression level

To elucidate the potential molecular mechanisms through which LINC01123 contributes to the progression of NSCLC, we explored the gene expression profiles under LINC01123 silencing condition. qRT-PCR, Western blot, and immunofluorescence showed that c-Myc mRNA and protein level was simultaneously upregulated in ectopic overexpression of LINC01123, while knockdown of LINC01123 could significantly reduce the expression level of c-Myc, both at mRNA and protein level (Fig. 5f–h). Furthermore, correlation analysis from GEPIA database revealed that there was a positive correlation between LINC01123 and c-Myc expression level (*R* = 0.37 and *P* < 0.001; Additional file 6: Figure S6A). Taken consideration of the above results, we supposed that LINC01123 could positively regulate c-Myc expression. To elucidate the potential molecular mechanisms through which LINC01123 contributes to regulate c-Myc expression, we first examined its localization in NSCLC cells, because the functions of lncRNA depended on its subcellular distribution [23]. Through FISH and subcellular fractionation assays, we identified that LINC01123 was mostly expressed in the cytoplasm (Additional file 5: Figure S5A-B).

### MiR-199a-5p is the intermediate downstream of LINC01123 that increases c-Myc level

**Fig. 6:**
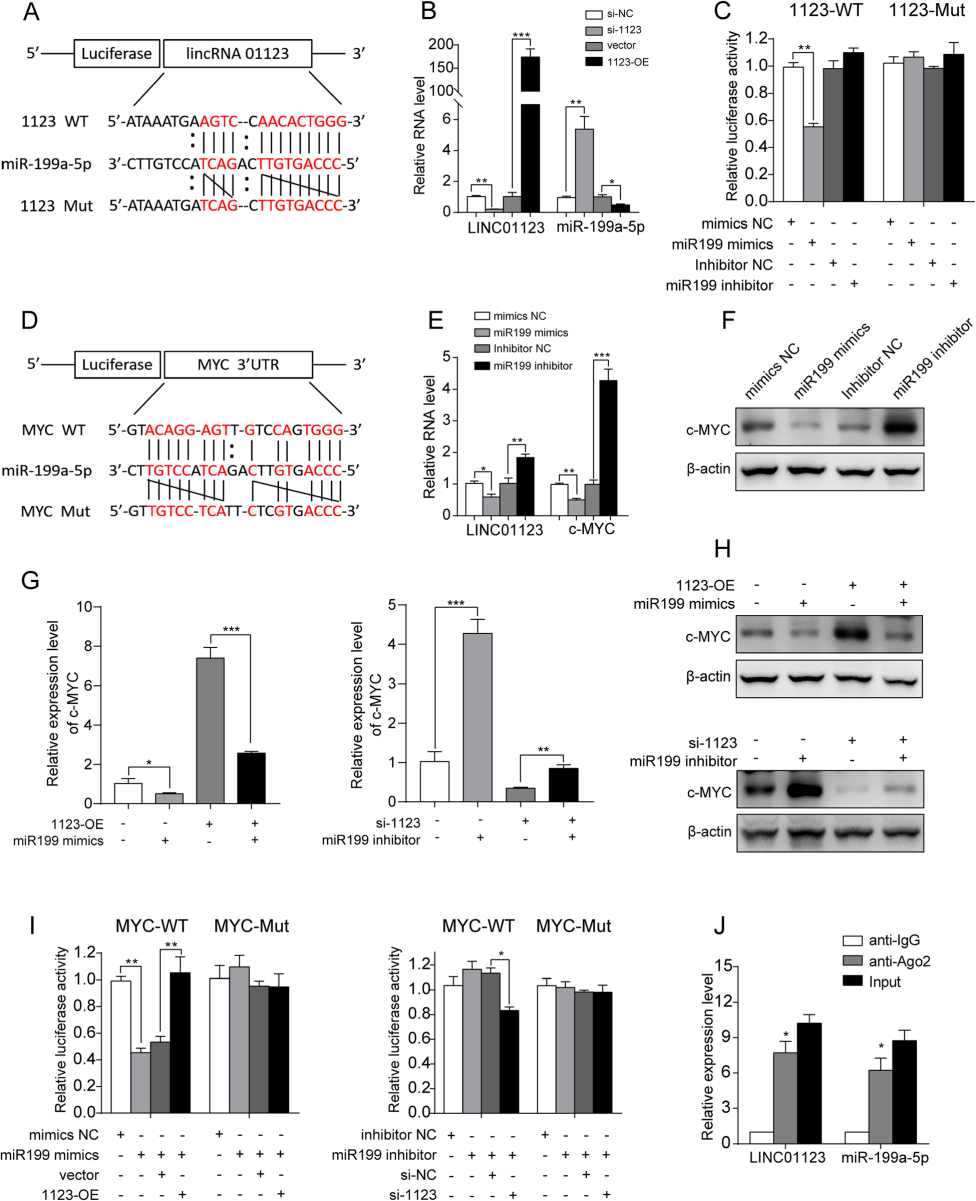
MiR-199a-5p is the intermediate downstream of LINC01123 that increases c-Myc level. **a** Schematic representation of the predicted binding site between miR-199a-5p and LINC01123 in wild-type and mutant-type was shown. **b** MiR-199a-5p mRNA level was negatively regulated by LINC01123 in A549 and H1299 cells. **c** The relative luciferase activity of 293 T cell was tested after co-transfection with LINC01123 wide-type and miR-199a-5p mimics. **d** Schematic representation of the predicted binding site between miR-199a-5p and c-Myc mRNA 3′-UTR in wild-type and mutant-type was shown. **e**, **f** LINC01123 mRNA level and c-Myc mRNA and protein levels were downregulated by miR-199a-5p mimics and upregulated by miR-199a-5p inhibitor in A549 and H1299 cells. **g**, **h** Rescue experiment of c-Myc mRNA and protein expression by co-transfection of 1123-OE pcDNA and miR-199a-5p mimics in A549 cell, or co-transfection of si-1123 and miR-199a-5p inhibitor in H1299 cell. **i** The dual-luciferase activity assay in 293 T cell demonstrated that miR-199a-5p mimics could significantly reduce the luciferase activity of wild-type but not mutant-type c-Myc 3′-UTR, and the reduction of luciferase activity could be restored by ectopic expression of LINC01123. MiR-199a-5p inhibitor slightly increased the luciferase activity of wild-type c-Myc 3′-UTR, but co-transfected with si-1123 reversed the luciferase activity. **j** A RIP assay was performed using anti-normal mouse IgG or anti-Ago2 in A549 cell lysates. The relative expression levels of LINC01123 and miR-199a-5p were detected by qRT-PCR. Data shown are mean ± SD (*n* = 3) (*P < 0.05, **P < 0.01, ***P < 0.001)

It was recently shown that cytoplasmic-localized lncRNAs can act as ceRNAs to regulate miRNAs [24]. To interpret the mechanism of how LINC01123 regulates the c-Myc level, we searched for partner molecules that could bind both LINC01123 and c-Myc. The miRNA target prediction bioinformatics (Starbase v3.0 and DIANA tools) suggested that both LINC01123 and c-Myc 3′UTR share a consensus binding site on miR-199a-5p, suggestive of a regulation mechanism among these three RNA molecules (Fig. 6a, d).

Considering LINC01123 has 13 putative miR-199a-5p complementary sites, it is reasonable to speculate that LINC01123 is able to efficiently inhibit miR-199a-5p function. We found that ectopic expression of LINC01123 decreased miR-199a-5p expression, which could be reversed by silencing of LINC01123 (Fig. 6b). To confirm the effect of miR-199a-5p on LINC01123 and c-Myc expression, A549 and H1299 cells were transfected with miR-199a-5p inhibitor and mimics, respectively. MiR-199a-5p mimics significantly inhibited LINC01123 and c-Myc expression, while miR-199a-5p inhibitor showed the opposite effect (Fig. 6e, f). Importantly, it was also found that simultaneous upregulation or downregulation of miR-199a-5p and LINC01123 greatly reversed LINC01123-mediated c-Myc expression to normal level at both mRNA and protein levels (Fig. 6g, h).

We further validated the direct binding of LINC01123 and 3′UTR of c-Myc mRNA with miR-199a-5p by dual luciferase reporter assay. The results demonstrated that miR-199a-5p mimics remarkably reduced the luciferase activities of the reporter plasmid containing the potential binding sequence of LINC01123 or 3′UTR of c-Myc mRNA (wild type, WT), but without obvious changes in the reporter plasmid containing mutated sequence (mutant type, MUT). Meanwhile, the miR-199a-5p inhibition could slightly increase the luciferase activity of wild-type LINC01123 or c-Myc 3′-UTR. To reinforce this conclusion, we performed an endogenous experiment. Co-transfection of LINC01123 could rescue the decreased luciferase activity of c-Myc (WT) treated with miR-199a-5p mimics. On the contrary, the luciferase activities of c-Myc (WT) enhanced by miR-199a-5p inhibition could be reversed by downregulation of LINC01123. These data illustrated that LINC01123 competitively decoyed miR-199a-5p and as a result regulated c-Myc mRNA expression level (Fig. 6c, i). According to the bioinformatics software, the sites of LINC01123/miR-199a-5p could also bind the Ago2 protein. So, RIP assay was performed in A549 and H1299 cell extracts utilizing the antibody against Ago2. LINC01123 and miR-199a-5p expression were detected by qRT-PCR. The results illustrated that both LINC01123 and miR-199a-5p were enriched in the Ago2 pellet relative to control IgG immunoprecipitate (Fig. 6j).

To verify the ceRNA network, miR-199a-5p expression level was also analyzed and Pearson analysis showed a negative correlation between the expression score of miR-199a-5p and LINC01123 (*R* = − 0.322 and *P* < 0.001; Additional file 6: Figure S6B). In addition, miR-199a-5p expressed much lower in NSCLC cells than in normal lung cells, while c-Myc expression was significantly higher in NSCLC cells than normal in lung cells, exhibiting similar expression tendency with LINC01123 (Additional file 6: Figure S6C-D).

### LINC01123 functions as an oncogene via miR-199a-5p and c-Myc

**Fig. 7:**
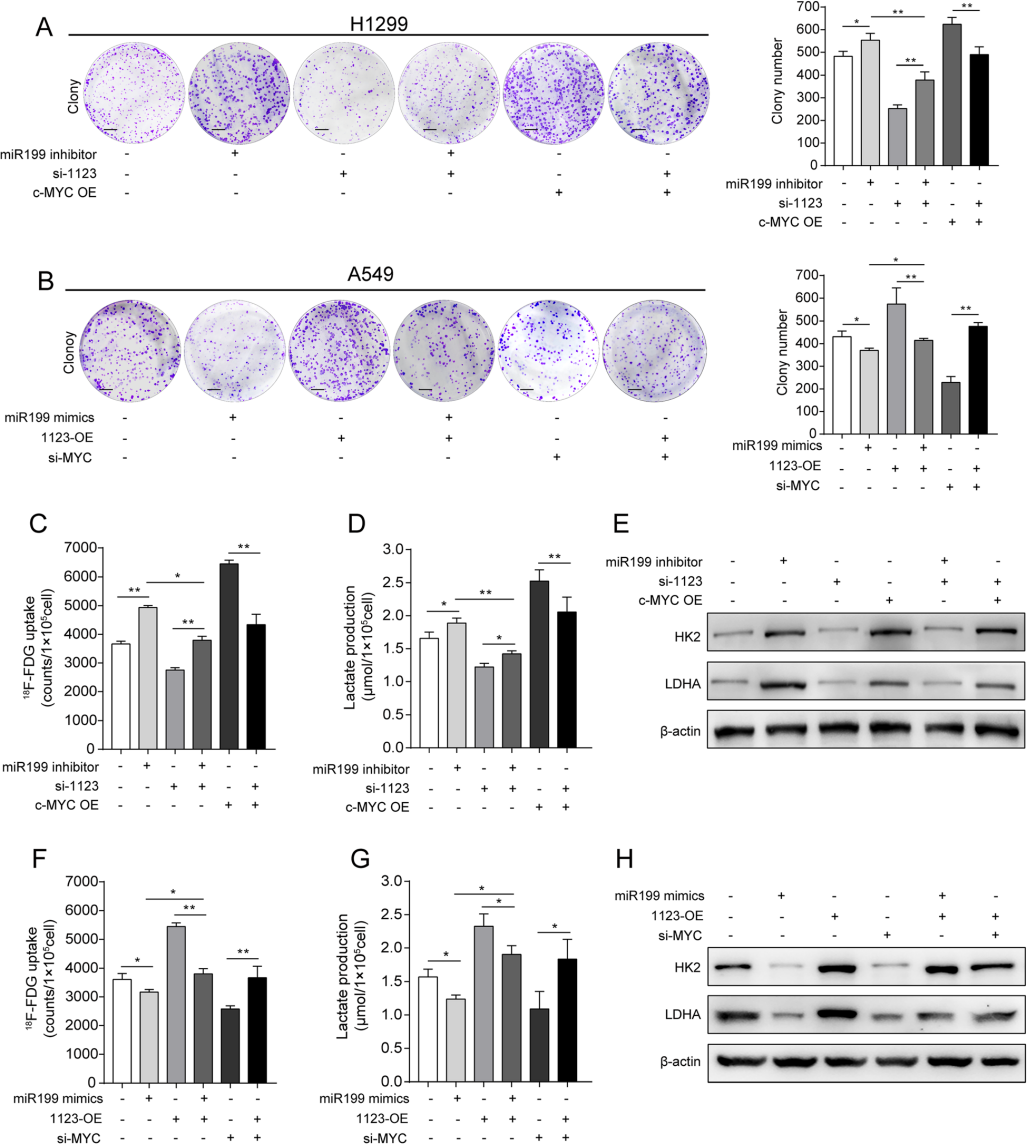
LINC01123 functions as an oncogene via miR-199a-5p and c-Myc. **a** Colony formation rescue experiment showed that cell proliferation reduced by si-1123 could be increased by miR-199a-5p inhibitor or Myc-OE in H1299 cell. **b** Colony formation rescue experiment showed that cell proliferation stimulated by ectopic expression of LINC01123 could be repressed by miR-199a-5p mimics or si-Myc in A549 cell. **c**–**e** Metabolic functional rescue experiment showed that ^18^F-FDG uptake, lactate production, and protein expression level of HK2 and LDHA reduced by si-1123 could be increased by miR-199a-5p inhibitor or Myc-OE in H1299 cell. **f**–**h** Metabolic functional rescue experiment showed that ^18^F-FDG uptake, lactate production, and protein expression level of HK2 and LDHA promoted by ectopic expression of LINC01123 could be repressed by miR-199a-5p mimics or si-Myc in A549 cell. Scale bar = 100 μm. Data shown are mean ± SD (*n* = 3) (*P < 0.05, **P < 0.01, ***P < 0.001)

We next explored whether oncogenic functions of LINC01123 depended on miR-199a-5p or c-Myc. Further functional study revealed that miR-199a-5p mimics or silencing of c-Myc suppressed the proliferation abilities of A549 cell stimulated by ectopic expression of LINC01123. On the contrary, miR-199a-5p inhibitor or c-Myc overexpression could rescue the ability of proliferation abilities of H1299 cell with knockdown of LINC01123 (Fig. 7a, b). MiR-199a-5p inhibitor or ectopic induction of c-Myc rescued the ^18^F-FDG uptake, lactate production, HK2, and LDHA protein expression decreased by LINC01123 knockdown, while induction of miR-199a-5p mimics or knockdown of c-Myc markedly reversed these biological effects under LINC01123 overexpression condition (Fig. 7c–h). All the above results indicated that LINC01123 functioned as an oncogene via miR-199a-5p and c-Myc.

## Discussion

Multiple evidences have verified that lncRNAs are aberrantly expressed in various tumor types, where they hold promise utilization for cancer diagnosis, monitoring, prognosis, or prediction for therapeutic responsiveness [18]. NSCLC is the most common type of lung cancer and is characterized by the dysregulation of gene networks including both protein-coding genes and non-coding RNAs [2]. To date, numerous lncRNAs are identified in NSCLC, presenting new perspectives for exploring molecular pathways in NSCLC pathogenesis [25]. In this study, we sought to search the aberrantly expressed lncRNAs by RNA-seq analysis of high ^18^F-FDG uptake NSCLC tissues. This current study found that LINC01123 was significantly upregulated in RNA-seq expression file and was associated with poor clinical outcomes in NSCLC patients, thus might represent as an independent prognostic biomarker in NSCLC.

As commonly recognized these years, cancer cells exhibit a unique metabolic phenotype with increased glucose uptake and lactate release to support their malignant biological processes [26]. Many studies have indicated that lncRNAs are intimately connected to the regulation of Warburg effect to support growth and survival of cancer cells [12, 27]. Elucidating the metabolic-related functions of lncRNAs provides a better understanding of the regulatory mechanisms of metabolism [28]. For example, lncRNA PCGEM1, by directly interacting with c-Myc and being a coactivator for c-Myc, functions as a master regulator of metabolic reprogramming in cancer [29]. Another notable example is lincRNA-p21, which is a hypoxia-responsive lncRNA. Being activated by HIF-1α, lincRNA-p21 in return stabilizes HIF-1α by disrupting the VHL-HIF-1α interaction, promoting glycolysis and OXPHOS downregulation [30]. Consistent with the previously reported lncRNAs, our study indicated that LINC01123 functioned as an oncogene by facilitating tumor malignant phenotype, as well as mediating energy status. It regulated metabolic adaptation by upregulating glycolytic gene expression and enzyme activity, thus promoting glycolysis in vitro and ^18^F-FDG uptake of subcutaneous xenograft in vivo.

c-Myc is a human oncogene and contributes to multiple hallmarks of cancer. As a transcriptional factor, early studies identify that c-Myc transcriptional targets are involved in many biological processes, such as metabolism, cell growth, cell cycle regulation, and apoptosis [31]. Besides a large number of protein-coding genes, many lncRNAs and microRNAs are newly proved downstream targets of c-Myc [32]. Lu et al. reported Myc targeted lncRNA DANCR, which was overexpressed in a variety of tumor types, could promote cancer cell proliferation [33]. LncRNA MINCR was another Myc-induced lncRNA able to modulate Myc’s transcriptional network in Burkitt lymphoma cells [34]. We here showed that LINC01123 was a novel transcript by c-Myc, which further participated in tumor malignant transforming processes. Our study thus expanded the breadth of transcriptional roles of c-Myc underlying glucose dependence of NSCLC.

The expression of c-Myc is under the tight control of many regulatory mechanisms, which is exquisitely regulated at the transcription, translation, protein stability, and activity levels. In recent years, it has become clear that lncRNAs add a crucial additional layer to the regulation of Myc and its downstream effects [35]. Zhang et al. reported that lncRNA-MIF (Myc inhibitory factor) had the ability to increase Fbxw7 mRNA expression, which was a characterized E3 ubiquitin ligase, thus causing c-Myc protein degradation [36]. To the best of our knowledge, gene modulation at mRNA level is a fast-acting strategy for cancer cells to adapt to susceptible environment and maximum cell survival [37]. LncRNA CCAT1 and SNHG3 were identified to modulate c-Myc mRNA expression via competing endogenous RNA (ceRNA) activity by sponging miR-155 or miR-182-5p, respectively [38, 39]. Here, we proved the ceRNA crosstalk network that LINC01123 served as a decoy to sequester miR-199a-5p from binding c-Myc mRNA, relieving its inhibitory effect on c-Myc expression. Rescue experiments indicated that LINC01123 functioned as an oncogene in promoting glycolysis as well as tumor growth through c-Myc-dependent pathway. Additionally, previous studies have revealed miR-199a-5p as a tumor suppressor in many tumor types, and it could suppress the Warburg effect by targeting HIF-1α [40–42]. In general, these results together illustrated the complicated and multi-dimensional interactions between LINC01123 and c-Myc, further elucidating the molecular mechanism of tumor progression and metabolic rewiring in NSCLC.

## Conclusion

**Fig. 8:**
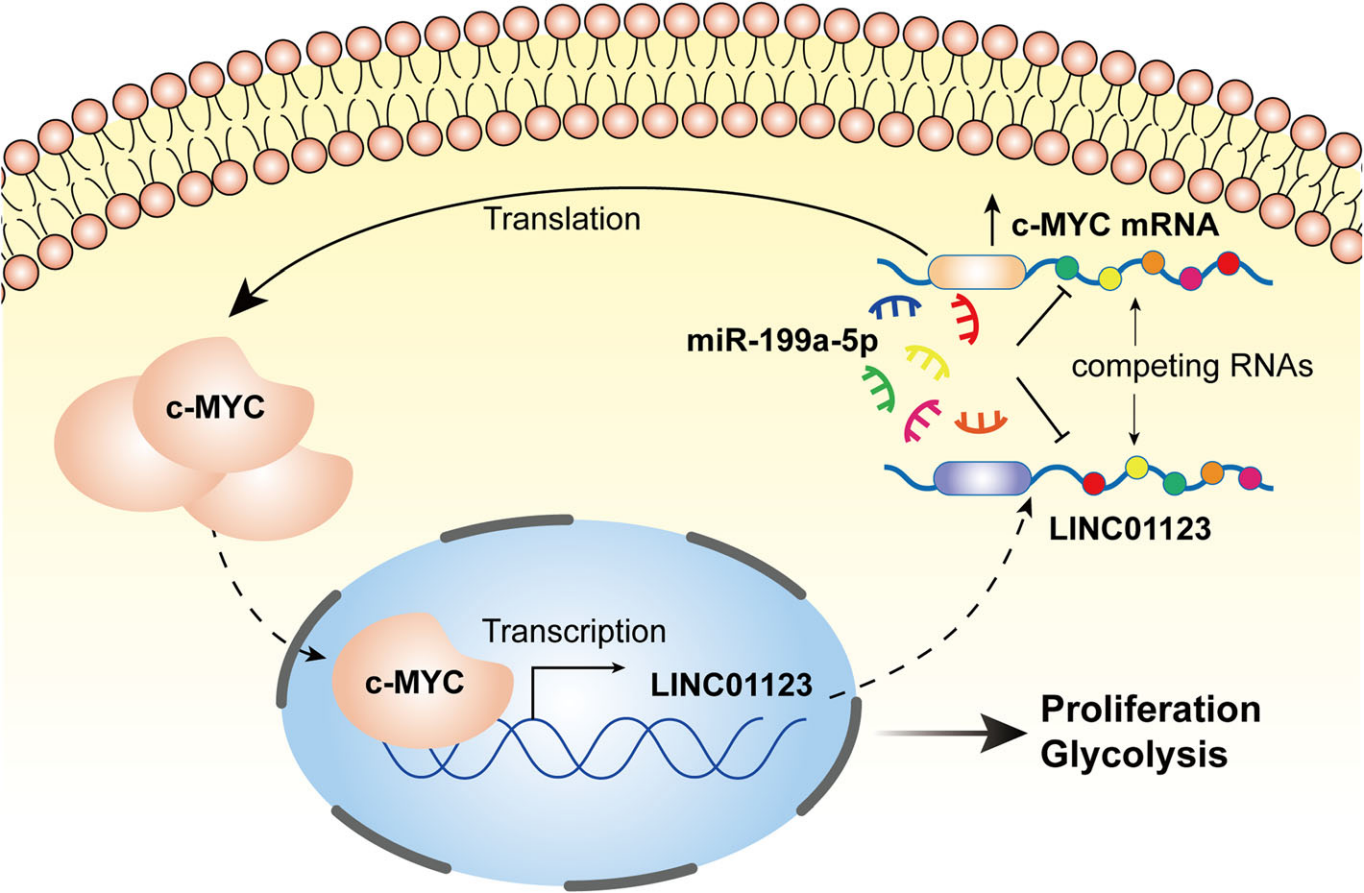
Schematic illustration of LINC01123 and c-Myc positive feedback loop in promoting proliferation and glycolysis

In summary, our study revealed that LINC01123 expression was upregulated in NSCLC tissues and cells. High expression of LINC01123 was associated with tumor progression and poor survival. LINC01123 was a direct transcription target of c-Myc and could in turn increase c-Myc expression. Consistent with the fact that c-Myc holds significant roles in driving metabolic adaptation, our findings suggest a model that LINC01123/c-Myc positive feedback loop facilitates tumor malignant progression and metabolic reprogramming (Fig. 8). This study offers insight into diversity and complexity of lncRNA-mRNA interaction and suggests that LINC01123 might be served as a new biomarker and therapeutic target of NSCLC.

## Additional files

Additional file 1: **Figure S1.** 18F-FDG PET/CT imaging and clinicopathologic features of three NSCLC patients enrolled in RNA-seq analysis. (DOCX 807 kb)

Additional file 2: **Figure S2.** The expressional profiles of genes in RNA-seq analysis. (DOCX 388 kb)

Additional file 3: **Figure S3.** The non-coding nature of LINC01123 was confirmed by coding-potential analysis. (DOCX 312 kb)

Additional file 4: **Figure S4.** LINC01123 expression is up-regulated in NSCLC. (DOCX 309 kb)

Additional file 5: **Figure S5.** LINC01123 mainly located at the cytoplasm. (DOCX 616 kb)

Additional file 6: **Figure S6.** The relationships between LINC01123, miR-199a-5p and c-Myc expression in NSCLC. (DOCX 292 kb)

Additional file 7: **Table S1.** Primers used in the paper. (DOCX 17 kb)

Additional file 8: **Table S2.** The top 10 upregulated and downregulated lncRNA/mRNAs in RNA-seq. (DOCX 22 kb)

## Data Availability

The datasets used and/or analyzed during the current study are available from the corresponding author on reasonable request.

## Abbreviations

ceRNA: Competing endogenous RNA
ChIP: Chromatin immunoprecipitation
CI: Confidence interval
ECAR: Extracellular acidification rate
FDG: Fluorodeoxyglucose
HR: Hazard ratio
IF: Immunofluorescence
IHC: Immunohistochemistry
ISH: In situ hybridization
lncRNA: Long non-coding RNA
ncRNAs: Non-coding RNAs
NSCLC: Non-small cell lung cancer
OCR: Oxygen consumption rate
OS: Overall survival
PET/CT: Positron emission tomography/computed tomography
RIP: RNA immunoprecipitation
SCLC: Small cell lung cancer
TNM: Tumor node metastasis staging system
