# Correction : LINC01123, a c-Myc-activated long non-coding RNA, promotes proliferation and aerobic glycolysis of non-small cell lung cancer through miR-199a-5p/c-Myc axis

**DOI:** 10.1186/s13045-023-01412-w

**Published:** 2023-02-18

**Authors:** Qian Hua, Mingming Jin, Baoming Mi, Fei Xu, Tian Li, Li Zhao, Jianjun Liu, Gang Huang

**Affiliations:** 1 Department of Nuclear Medicine, Renji Hospital, School of Medicine, Shanghai Jiaotong University, Shanghai, 200127 China; 2 Shanghai Key Laboratory of Molecular Imaging, Shanghai University of Medicine and Health Sciences, Shanghai, 201318 China; 3 Department of Nuclear Medicine, Affiliated Hospital of Jiangnan University (Wuxi 4th People’s Hospital), Wuxi, 214062 Jiangsu China

**Correction : Journal of Hematology & Oncology** **https://doi.org/10.1186/s13045-019-0773-y**

The original version of this article unfortunately contained a mistake in Fig. 7A, B. In Fig. 7A, the first, third and fourth colony was previously used by mistake with Fig. 2E. In Fig. 7B, the fourth colony was used by mistake with the fifth colony. The revised corrected Fig. 7A, , B is given below.

**Fig. 7:**
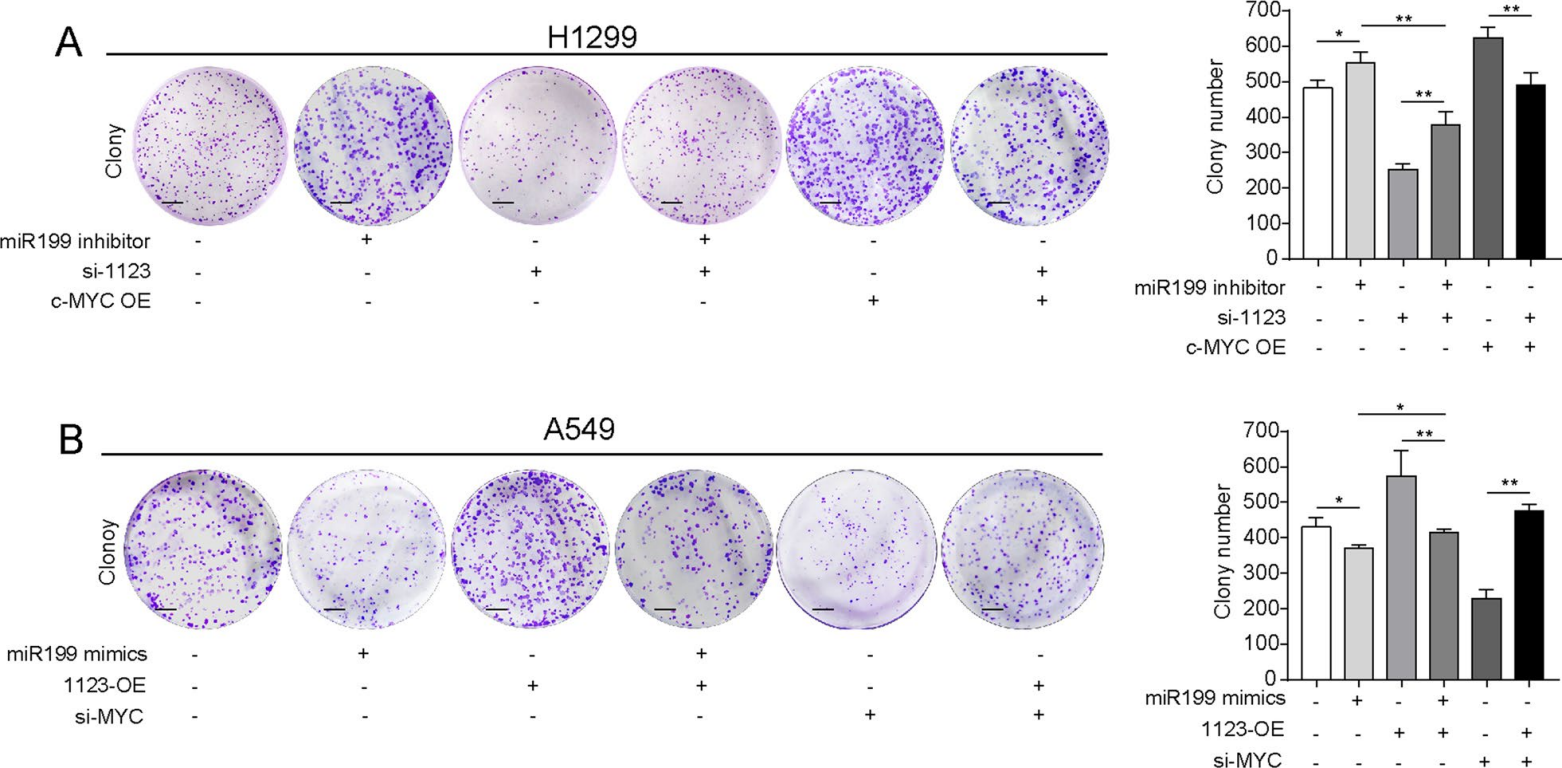
LINC01123 functions as an oncogene via miR-199a-5p and c-Myc. **a** Colony formation rescue experiment showed that cell proliferation reduced by si-1123 could be increased by miR-199a-5p inhibitor or Myc-OE in H1299 cell. **b** Colony formation rescue experiment showed that cell proliferation stimulated by ectopic expression of LINC01123 could be repressed by miR-199a-5p mimics or si-Myc in A549 cell. **c**–**e** Metabolic functional rescue experiment showed that ^18^F-FDG uptake, lactate production, and protein expression level of HK2 and LDHA reduced by si-1123 could be increased by miR-199a-5p inhibitor or Myc-OE in H1299 cell. **f**–**h** Metabolic functional rescue experiment showed that.^18^F-FDG uptake, lactate production, and protein expression level of HK2 and LDHA promoted by ectopic expression of LINC01123 could be repressed by miR199a-5p mimics or si-Myc in A549 cell. Scale bar = 100 μm. Data shown are mean ± SD (n = 3) (*P < 0.05, **P < 0.01, ***P < 0.001)

