# Supplementary material for: LINC01123, a c-Myc-activated long non-coding RNA, promotes proliferation and aerobic glycolysis of non-small cell lung cancer through miR-199a-5p/c-Myc axis
Source: J Hematol Oncol. 2019 Sep 5;12:91. doi: 10.1186/s13045-019-0773-y (PMC6728969; doi:10.1186/s13045-019-0773-y)
Supplement: Supplementary file 1 — Figure S1. 18F-FDG PET/CT imaging and clinicopathologic features of three NSCLC patients enrolled in RNA-seq analysis. (DOCX 807 kb) [file 13045_2019_773_MOESM1_ESM.docx]

**
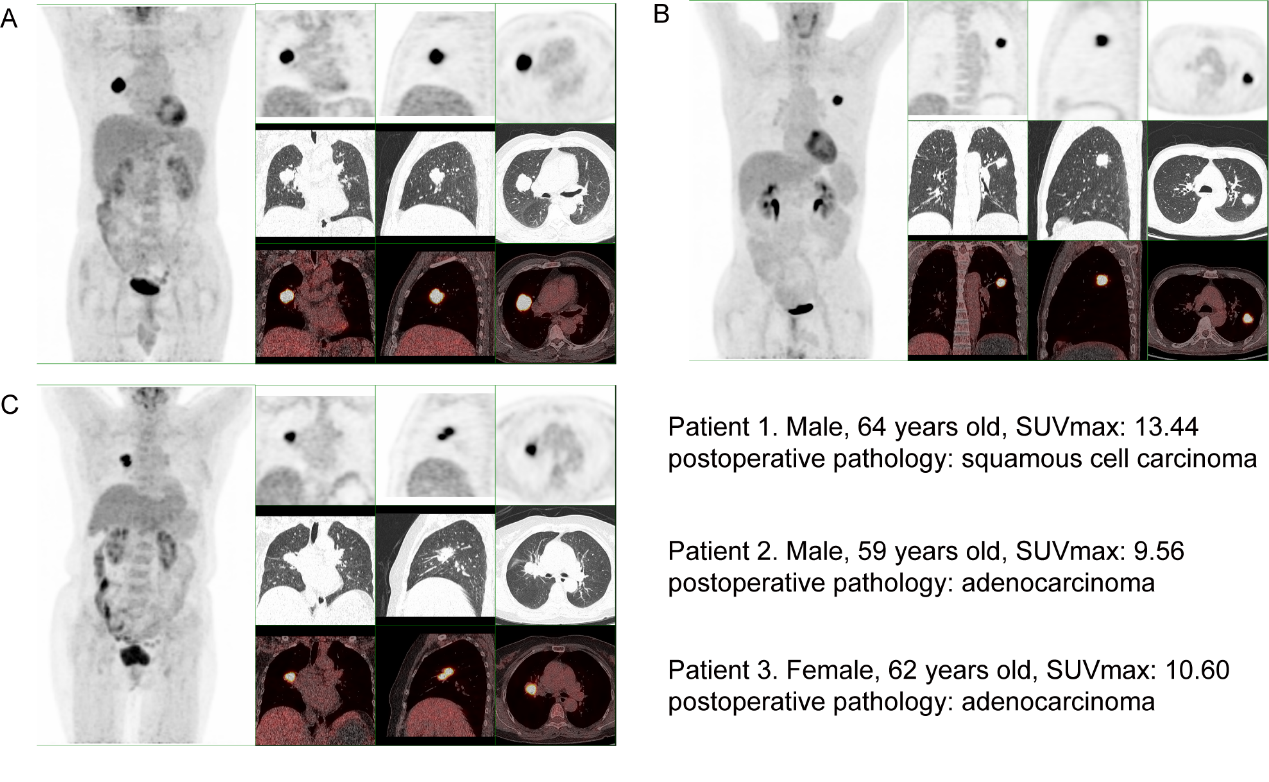
**

**Figure S1. ^18^F-FDG PET/CT imaging and clinicopathologic features of three NSCLC patients enrolled in RNA-seq analysis.**
