# Supplementary material for: LINC01123, a c-Myc-activated long non-coding RNA, promotes proliferation and aerobic glycolysis of non-small cell lung cancer through miR-199a-5p/c-Myc axis
Source: J Hematol Oncol. 2019 Sep 5;12:91. doi: 10.1186/s13045-019-0773-y (PMC6728969; doi:10.1186/s13045-019-0773-y)
Supplement: Supplementary file 2 — Figure S2. The expressional profiles of genes in RNA-seq analysis. (DOCX 388 kb) [file 13045_2019_773_MOESM2_ESM.docx]

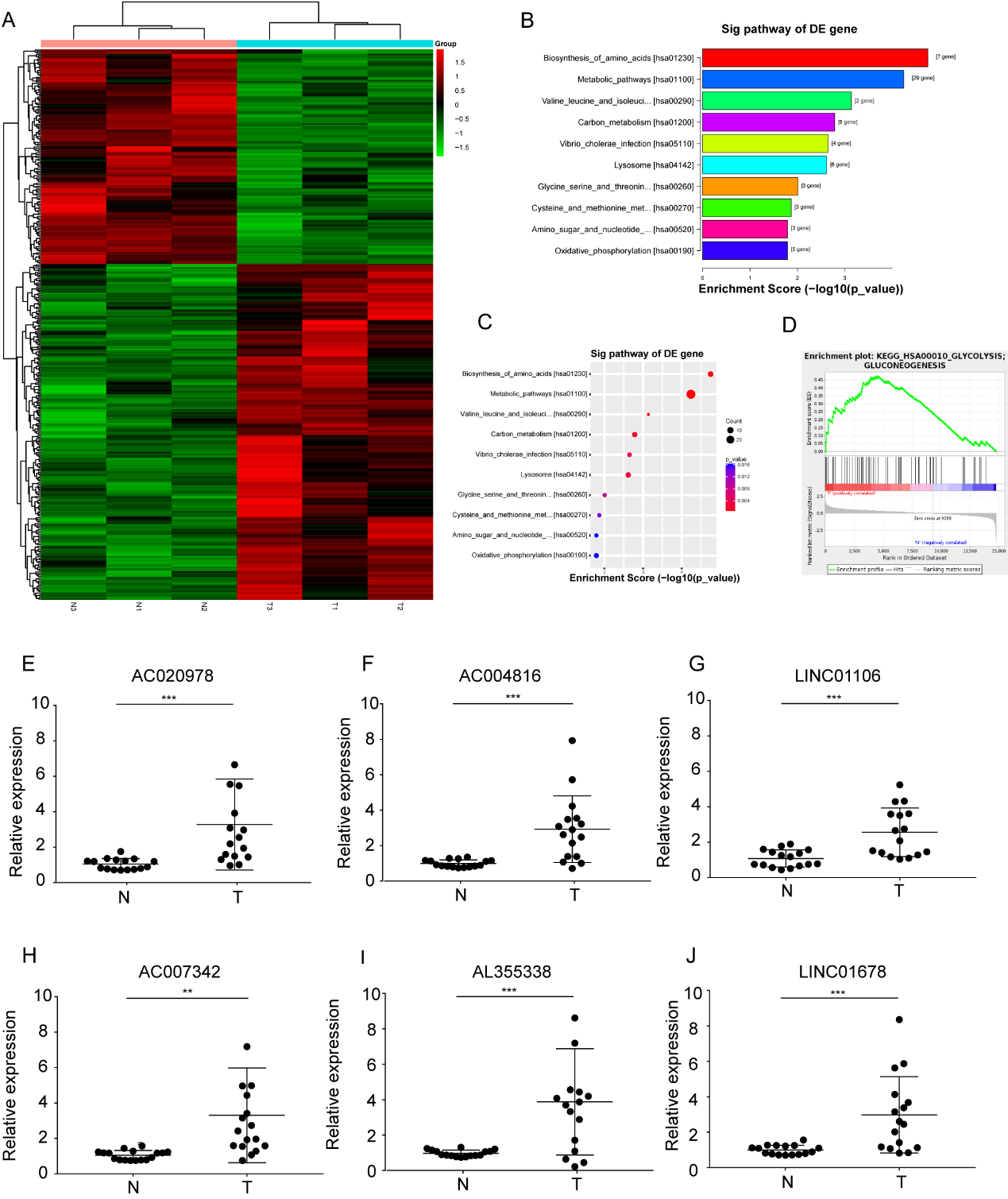


**Figure S2. The expressional profiles of genes in RNA-seq analysis.**

(A) Hierarchical clustering analysis of differentially expressed genes in RNA-seq analysis.

(B-D) GSEA and KEGG pathway analysis showing that the top 10 overexpressed lncRNA and mRNAs were enriched in metabolic pathways, especially in glycolysis pathway.

(E-J) The expression level of 6 random selected upregulated lncRNAs in 16 paired NSCLC and adjacent normal tissues by qRT-PCR. Data shown are mean±SD (n = 3). (*P < 0.05, **P < 0.01, ***P < 0.001)
