# Supplementary material for: LINC01123, a c-Myc-activated long non-coding RNA, promotes proliferation and aerobic glycolysis of non-small cell lung cancer through miR-199a-5p/c-Myc axis
Source: J Hematol Oncol. 2019 Sep 5;12:91. doi: 10.1186/s13045-019-0773-y (PMC6728969; doi:10.1186/s13045-019-0773-y)
Supplement: Supplementary file 3 — Figure S3. The non-coding nature of LINC01123 was confirmed by coding-potential analysis. (DOCX 312 kb) [file 13045_2019_773_MOESM3_ESM.docx]

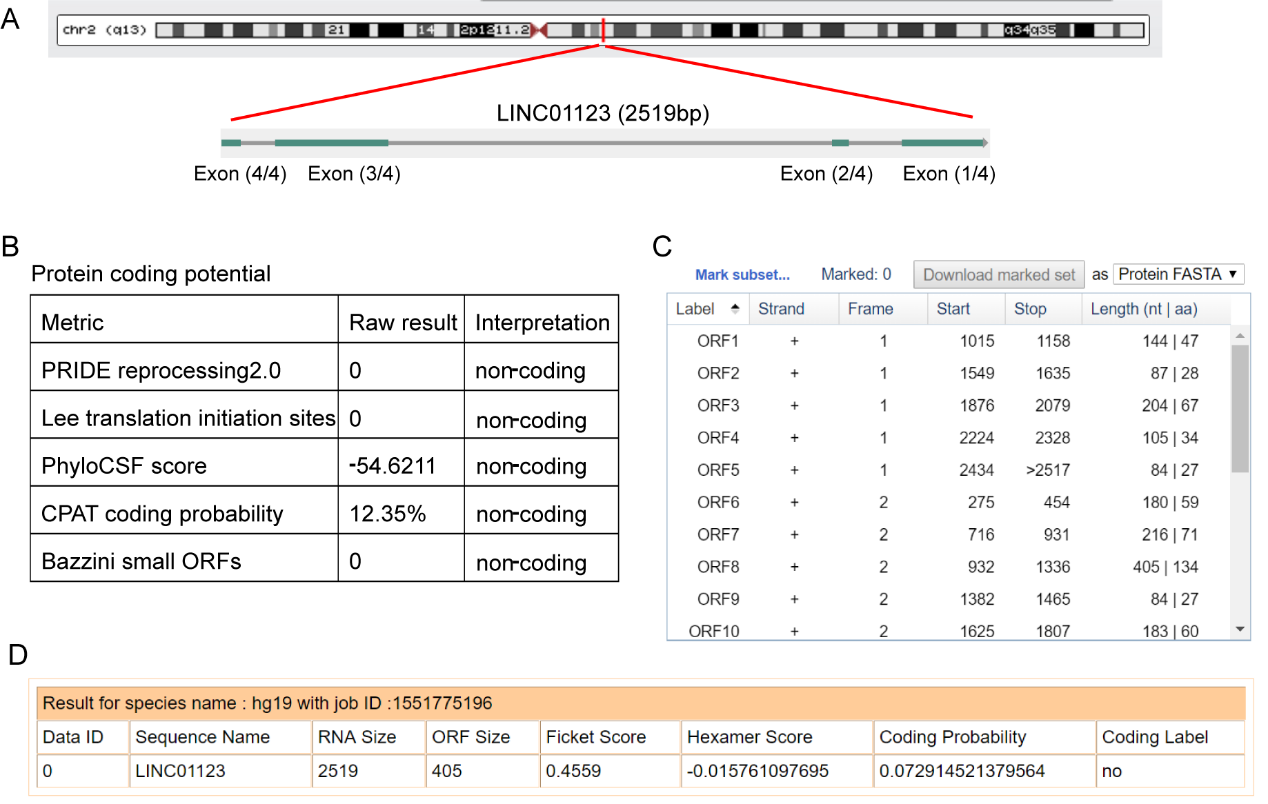


**Figure S3. The non-coding nature of LINC01123 was confirmed by coding-potential analysis.**

(A) LINC01123 is located on chromosome 2 in humans and composed of four exons with a full length of 2519 bp. (B, D) The coding potential of LINC01123 in several prediction softwares, and results showed that LINC01123 didn’t have any coding potential. (C) ORF Finder software prediction (https://www.ncbi.nlm.nih.gov/orffinder/) for the protein-coding potential of LINC01123.
