# Supplementary material for: LINC01123, a c-Myc-activated long non-coding RNA, promotes proliferation and aerobic glycolysis of non-small cell lung cancer through miR-199a-5p/c-Myc axis
Source: J Hematol Oncol. 2019 Sep 5;12:91. doi: 10.1186/s13045-019-0773-y (PMC6728969; doi:10.1186/s13045-019-0773-y)
Supplement: Supplementary file 4 — Figure S4. LINC01123 expression is up-regulated in NSCLC. (DOCX 309 kb) [file 13045_2019_773_MOESM4_ESM.docx]

**
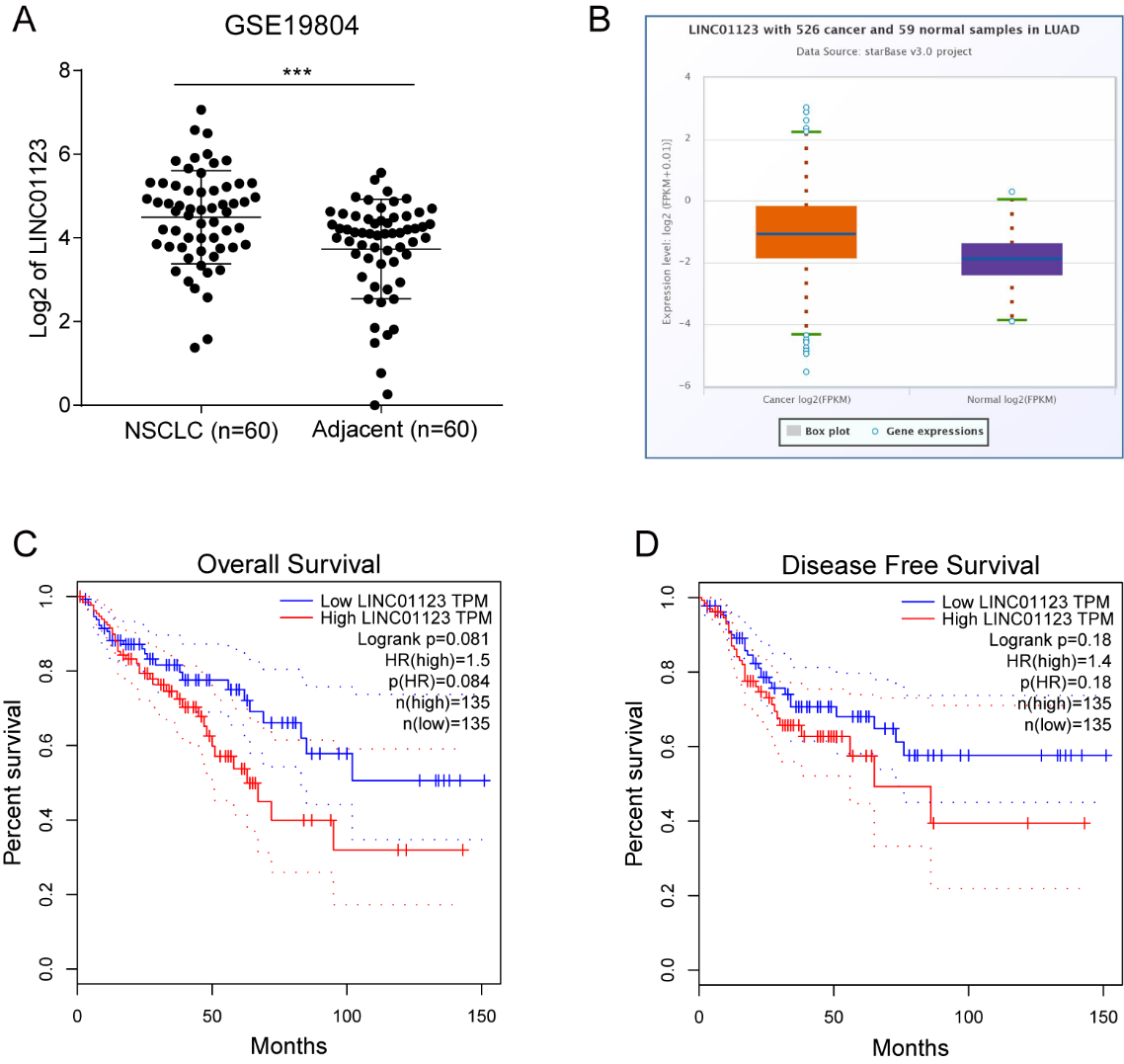
**

**Figure S4. LINC01123 expression is up-regulated in NSCLC.**

(A) GEO datasets (GSE19804) from R2: Genomics Analysis and Visualization Platform (http://r2.amc.nl) indicated that LINC01123 expression was prominently higher in NSCLC tissues compared to normal lung tissues. P < 0.001 by Student’s t-test.

(B) The expression pattern of LINC01123 in lung adenocarcinoma based on TCGA data from starBase V3.0 (http://starbase.sysu.edu.cn).

(C-D) TCGA data from GEPIA Platform (http://gepia.cancer-pku.cn/) further demonstrated that high LINC01123 expression indicated poor survival of NSCLC patients. The median expression level of LINC01123 was used as the cut-off.
