# Supplementary material for: LINC01123, a c-Myc-activated long non-coding RNA, promotes proliferation and aerobic glycolysis of non-small cell lung cancer through miR-199a-5p/c-Myc axis
Source: J Hematol Oncol. 2019 Sep 5;12:91. doi: 10.1186/s13045-019-0773-y (PMC6728969; doi:10.1186/s13045-019-0773-y)
Supplement: Supplementary file 5 — Figure S5. LINC01123 mainly located at the cytoplasm. (DOCX 616 kb) [file 13045_2019_773_MOESM5_ESM.docx]

**
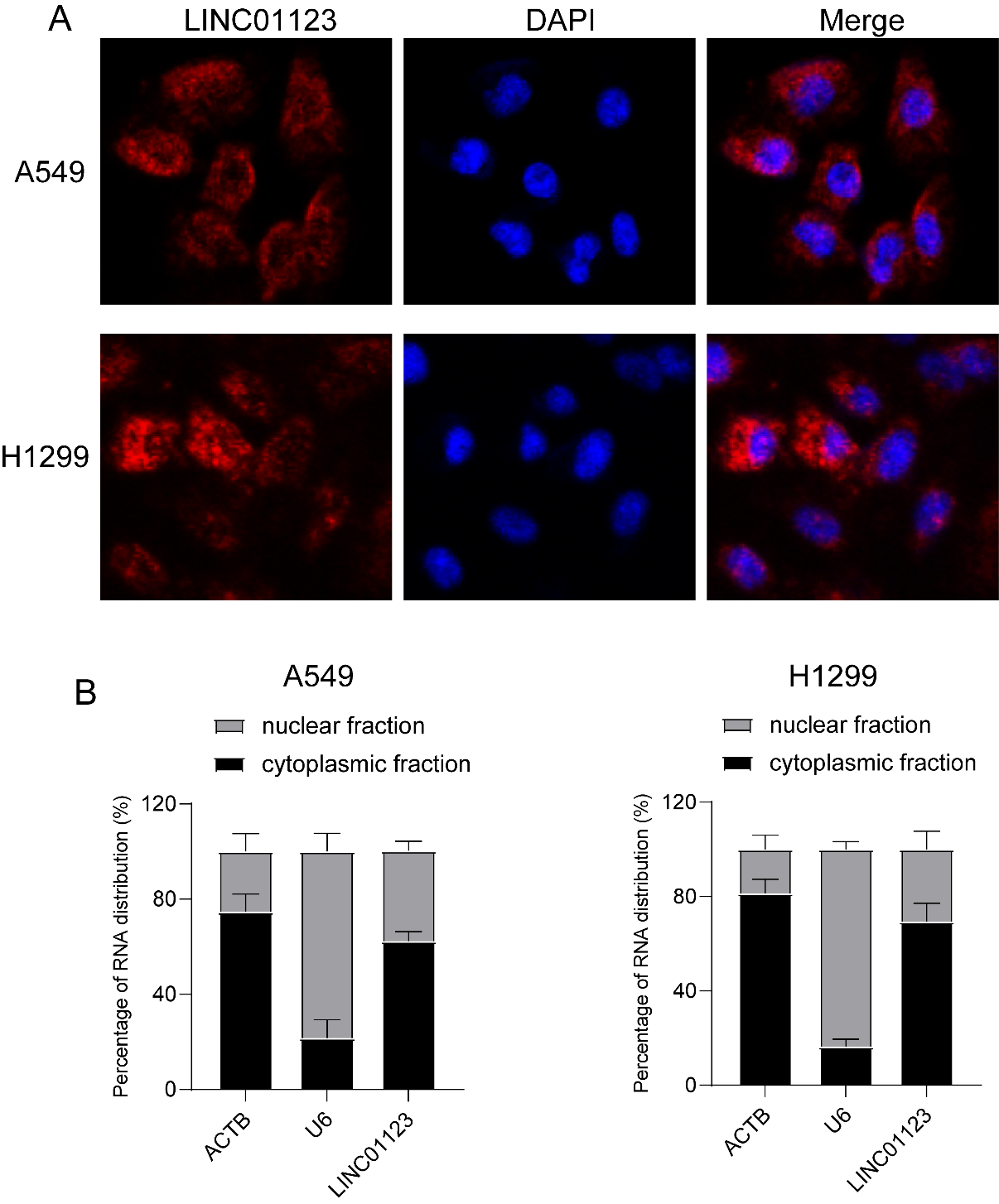
**

**Figure S5. LINC01123 mainly located at the cytoplasm.**

(A) Representive FISH images showed the expression of LINC01123 in A549 and H1299 cells (red). Nuclei were stained by DAPI (blue).

(B) Relative LINC01123 expression levels in nuclear and cytosolic fractions of A549 and H1299 cells. Nuclear controls: U6, cytosolic controls: ACTB.
