# Supplementary material for: LINC01123, a c-Myc-activated long non-coding RNA, promotes proliferation and aerobic glycolysis of non-small cell lung cancer through miR-199a-5p/c-Myc axis
Source: J Hematol Oncol. 2019 Sep 5;12:91. doi: 10.1186/s13045-019-0773-y (PMC6728969; doi:10.1186/s13045-019-0773-y)
Supplement: Supplementary file 6 — Figure S6. The relationships between LINC01123, miR-199a-5p and c-Myc expression in NSCLC. (DOCX 292 kb) [file 13045_2019_773_MOESM6_ESM.docx]

**
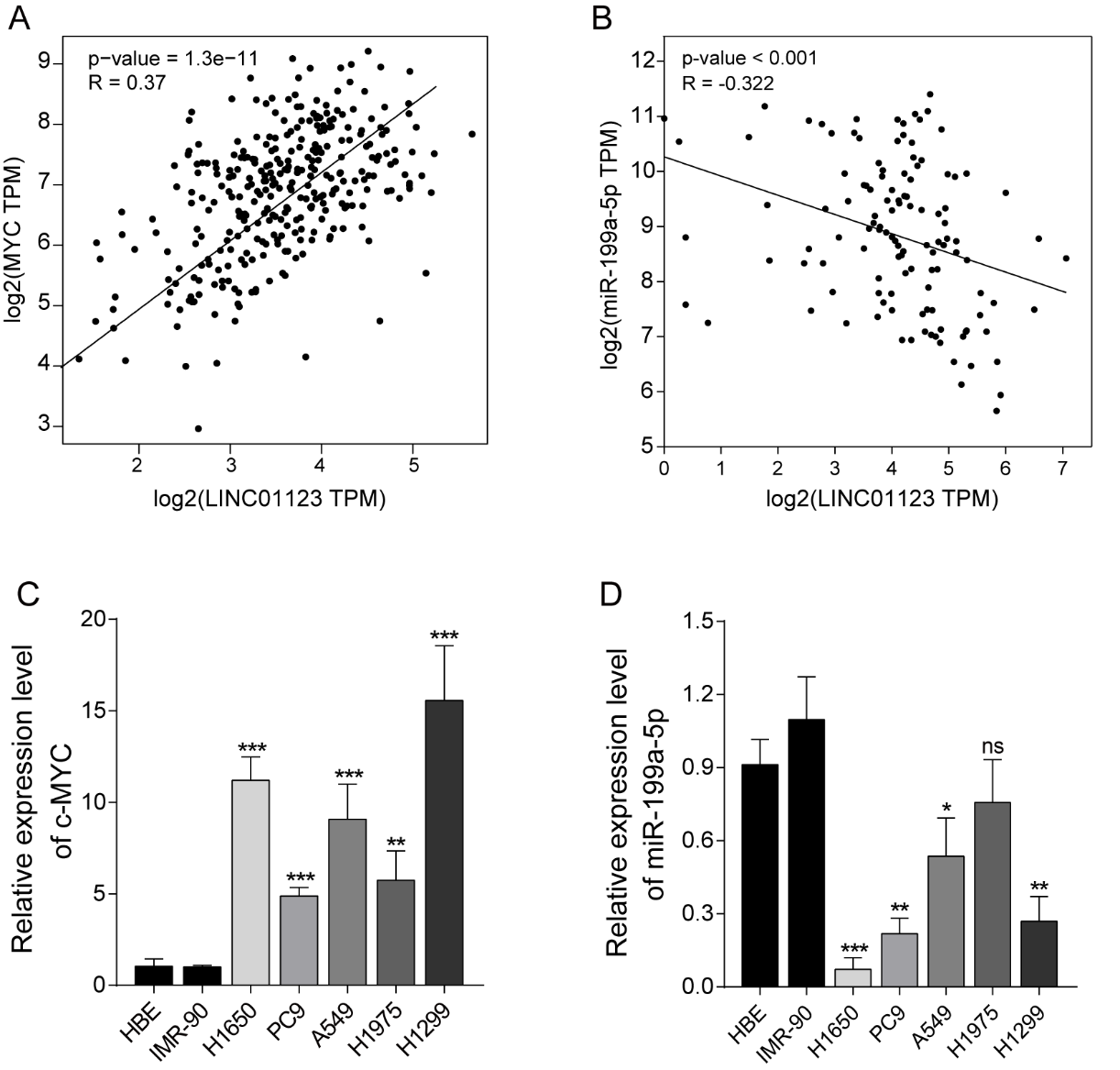
**

**Figure S6. The relationships between LINC01123, miR-199a-5p and c-Myc expression in NSCLC.**

(A) A positive correlation between LINC01123 and c-Myc expression was observed based on TCGA database from GEPIA Platform (http://gepia.cancer-pku.cn/). R = 0.37, P <0.001 by Spearman correlation test.

(B) LINC01123 was inversely correlated with miR-199a-5p expression in NSCLC tissues based on TCGA database from R2 Platform (http://r2.amc.nl). R = -0.322, P <0.001 by Spearman correlation test.

(C-D) QRT-PCR analysis of c-Myc and miR-199a-5p expression in the NSCLC cell lines.
