# Supplementary material for: LINC01123, a c-Myc-activated long non-coding RNA, promotes proliferation and aerobic glycolysis of non-small cell lung cancer through miR-199a-5p/c-Myc axis
Source: J Hematol Oncol. 2019 Sep 5;12:91. doi: 10.1186/s13045-019-0773-y (PMC6728969; doi:10.1186/s13045-019-0773-y)
Supplement: Supplementary file 7 — Table S1. Primers used in the paper. (DOCX 17 kb) [file 13045_2019_773_MOESM7_ESM.docx]

**Table S1. Primers used in the paper were listed:**

| **Gene** | **Primer** | **Sequence(5′-3′)** |
| --- | --- | --- |
| **Primers for qRT-PCR** | | |
| ACTB | forward | CATGTACGTTGCTATCCAGGC |
|  | reverse | CTCCTTAATGTCACGCACGAT |
| LINC01123 | forward | ACAGTGGCCGCACGCATAGCTG |
|  | reverse | CTGACGACCGAGGTGACAACGATGA |
| AC004816 | forward | GCCTTGGCTTCCCAAAGTGCTG |
|  | reverse | CAAACGGCCACAGCCCACG |
| AC020978 | forward | GGACCATGCAGTTTTGTGGAAGA |
|  | reverse | GGCAGAGGCAGGTGGATCAC |
| LINC01106 | forward | GGAACGACTGAGACGGCTAAAC |
|  | reverse | CATGTCTGTTGAAACAAGGCAGC |
| AC007342 | forward | CCAACCTCAGAGGCAGCTTCAAGTG |
|  | reverse | TAGCTGGGCACAGTGTTCACACCTG |
| LINC01678 | forward | GGACTGCAATGGCCTCCCACAC |
|  | reverse | CTGGGCTTGTCCTTGGAGGAAGT |
| AL355338 | forward | CAGAGAGAGGAACTGAGAGGAGAAGGAG |
|  | reverse | TCCTACTTCTGGGATCGGTTCCTGG |
| c-MYC | forward | AGCGACTCTGAGGAGGAAC |
|  | reverse | TGTGAGGAGGTTTGCTGTG |
| GLUT1 | forward | ATGAACTACCCTCACTCCAGC |
|  | reverse | TATTGGACACAGCTTGGATGCC |
| LDHA | forward | AGGAGAAACACGCCTTGATTTAG |
|  | reverse | ACGAGCAGAGTCCAGATTACAA |
| PKM2 | forward | GGGCCATAATCGTCCTCACC |
|  | reverse | TTGCACAGCACAGGGAAGAT |
| HK2 | forward | GAATGGGAAGTGGGGTGGAG |
|  | reverse | GAGGAGGATGCTCTCGTCCA |
| PDK1 | forward | GGTGTTTACCCCCCTATTCAAG |
|  | reverse | CGGGAGGTCTCAACACGA |
| G6PDH | forward | TGGAGATCATCATGAAAGAGACC |
|  | reverse | GCGAATGACACCGTACTCCT |
| PFKL | forward | CTGGCTGCTACGTGAGAACA |
|  | reverse | GCTTCTCATCCGCTCTCCAG |
| U6 | forward | CTCGCTTCGGCAGCACA |
|  | reverse | CTCAACTGGTGTCGTGGA |
| miR-199a-5p | forward | GCCGAGCCCAGTGTTCAGACT |
|  | reverse | CTCAACTGGTGTCGTGGA |
| **Primers for stem-loop RT-PCR of miRNAs** | | |
| U6 | RT | AACGCTTCACGAATTTGCGT |
| miR-199a-5p | RT | CTCAACTGGTGTCGTGGAGTCGGCAATTCAGTTGAGGAACAGGT |
| **Sequences for gene knockdown** | | |
| si-1123-1 | forward | CUGAACGUCUUGCAACAGUTT |
|  | reverse | ACUGUUGCAAGACGUUCAGTT |
| si-1123-2 | forward | GCCCUAGGAAAUCCGUAAUTT |
|  | reverse | AUUACGGAUUUCCUAGGGCTT |
| si-c-MYC | forward | CCUGAGACAGAUCAGCAACAATT |
|  | reverse | UUGUUGCUGAUCUGUCUCAGGTT |
| **Primers for ChIP-qPCR** | | |
| ChIP-1123 promoter 1-1 | forward | CCTGAGTAGCTTGGAATACGG |
|  | reverse | CGGATCACTTGAGGTCAGG |
| ChIP-1123 promoter 1-2 | forward | TCGAGGGTCACTGCAACC |
|  | reverse | GGTCAGGAGTTCAAGACAAGC |
| ChIP-1123 promoter 2-1 | forward | GAATACGGGCACCCACCA |
|  | reverse | TCGCAGCAATTTGAGAGGC |
| ChIP-1123 promoter 2-2 | forward | TCCGTCATCTCTGGGACCT |
|  | reverse | TGGACTAAGGTGAGCCACTTC |

**Sequences of probes for LINC01123 RNA FISH**

5’-DIG- CCCTTGCATGGTACACTTGAAATAGATGGGCTGGA-DIG-3’
