# Supplementary material for: LINC01123, a c-Myc-activated long non-coding RNA, promotes proliferation and aerobic glycolysis of non-small cell lung cancer through miR-199a-5p/c-Myc axis
Source: J Hematol Oncol. 2019 Sep 5;12:91. doi: 10.1186/s13045-019-0773-y (PMC6728969; doi:10.1186/s13045-019-0773-y)
Supplement: Supplementary file 8 — Table S2. The top 10 upregulated and downregulated lncRNA/mRNAs in RNA-seq. (DOCX 22 kb) [file 13045_2019_773_MOESM8_ESM.docx]

**Table S2. The top 10 upregulated and downregulated lncRNA/mRNAs in RNA-seq.**

**Upregulated lncRNAs**

| **Track_id** | **Gene_Name** | **Gene_Type** | **log2FC** | **Fold_Change** | **p_value** | **q_value** |
| --- | --- | --- | --- | --- | --- | --- |
| ENSG00000274605.1_3 | AL355338.1 | lincRNA | 1.358995395 | 2.565065019 | 0.029335933 | 0.910360898 |
| ENSG00000259797.1_4 | AC020978.2 | sense_intronic | 1.092494508 | 2.132424271 | 0.017528656 | 0.873750285 |
| ENSG00000259153.1_3 | AC004816.1 | lincRNA | 1.012161264 | 2.016930349 | 0.025503712 | 0.903875729 |
| ENSG00000204588.5_2 | LINC01123 | lincRNA | 0.921317112 | 1.893843493 | 0.012589098 | 0.814804365 |
| ENSG00000261804.1_3 | AC007342.4 | lincRNA | 0.909315651 | 1.878154375 | 0.008176926 | 0.807190972 |
| ENSG00000175772.10_2 | LINC01106 | lincRNA | 0.847449821 | 1.799317549 | 0.014610302 | 0.853286366 |
| ENSG00000265800.1_4 | AC022211.3 | sense_intronic | 0.815701994 | 1.760154409 | 0.022348931 | 0.890442684 |
| ENSG00000253838.1_4 | AC007991.2 | sense_intronic | 0.78444159 | 1.722425507 | 0.044740969 | 0.957531811 |
| ENSG00000260597.1_4 | AC012531.1 | lincRNA | 0.775087632 | 1.711293995 | 0.044878657 | 0.958078019 |
| ENSG00000225331.1_2 | LINC01678 | lincRNA | 0.767075112 | 1.701816059 | 0.024610963 | 0.903875729 |

**Downregulated lncRNAs**

| **Track_id** | **Gene_Name** | **Gene_Type** | **log2FC** | **Fold_Change** | **p_value** | **q_value** |
| --- | --- | --- | --- | --- | --- | --- |
| ENSG00000239268.2_4 | AC092691.1 | lincRNA | -1.502591253 | 0.352918936 | 0.02228273 | 0.890442684 |
| ENSG00000273760.1_3 | AC245041.1 | lincRNA | -0.949571967 | 0.517786061 | 0.040608844 | 0.943535863 |
| ENSG00000273186.1_4 | AL359091.4 | sense_intronic | -0.909416473 | 0.532400388 | 0.000424925 | 0.345786603 |
| ENSG00000224239.1_3 | AC090044.1 | lincRNA | -0.906861857 | 0.533343958 | 0.031993368 | 0.925987581 |
| ENSG00000226276.1_2 | AC093382.1 | lincRNA | -0.829869332 | 0.562580194 | 0.016104657 | 0.867578464 |
| ENSG00000255864.5_3 | AC069208.1 | lincRNA | -0.740335822 | 0.598599997 | 0.003901588 | 0.65397486 |
| ENSG00000277135.1_4 | AC012409.3 | lincRNA | -0.712527189 | 0.610250218 | 0.011047987 | 0.812222054 |
| ENSG00000248476.1_3 | BACH1-IT1 | sense_intronic | -0.668503372 | 0.629159028 | 0.027909989 | 0.905212674 |
| ENSG00000249621.1_3 | AC113349.1 | lincRNA | -0.652031861 | 0.636383413 | 0.0408065 | 0.943535863 |
| ENSG00000271643.1_3 | AC112220.4 | lincRNA | -0.622031261 | 0.649755452 | 0.032875527 | 0.934507017 |

**Upregulated mRNAs**

| **Track_id** | **Gene_Name** | **Gene_Type** | **log2FC** | **Fold_Change** | **p_value** | **q_value** |
| --- | --- | --- | --- | --- | --- | --- |
| ENSG00000160862.12_2 | AZGP1 | protein_coding | 3.085417401 | 8.487957313 | 0.010576012 | 0.812222054 |
| ENSG00000124664.10_2 | SPDEF | protein_coding | 2.204100241 | 4.607870721 | 0.004051272 | 0.657800093 |
| ENSG00000206053.12_4 | JPT2 | protein_coding | 2.182358075 | 4.538948362 | 0.000796061 | 0.382770221 |
| ENSG00000114631.10_3 | PODXL2 | protein_coding | 2.144462186 | 4.421274125 | 0.008794513 | 0.807190972 |
| ENSG00000130203.9_3 | APOE | protein_coding | 2.065204728 | 4.184933586 | 0.010711767 | 0.812222054 |
| ENSG00000146070.16_2 | PLA2G7 | protein_coding | 1.846280536 | 3.595719636 | 0.003546267 | 0.629366242 |
| ENSG00000152766.5_2 | ANKRD22 | protein_coding | 1.777946151 | 3.429376141 | 0.018678915 | 0.886428701 |
| ENSG00000165215.6_3 | CLDN3 | protein_coding | 1.756262753 | 3.378218756 | 0.033495022 | 0.937451129 |
| ENSG00000140297.12_3 | GCNT3 | protein_coding | 1.696501976 | 3.241141431 | 0.02214566 | 0.890442684 |
| ENSG00000171155.7_2 | C1GALT1C1 | protein_coding | 1.635336391 | 3.106599777 | 0.00574835 | 0.758958248 |

**Downregulated mRNAs**

| **Track_id** | **Gene_Name** | **Gene_Type** | **log2FC** | **Fold_Change** | **p_value** | **q_value** |
| --- | --- | --- | --- | --- | --- | --- |
| ENSG00000149021.6_3 | SCGB1A1 | protein_coding | -4.395198717 | 0.04752404 | 0.042268315 | 0.948649247 |
| ENSG00000124237.5_2 | C20orf85 | protein_coding | -2.254482396 | 0.209571959 | 0.00015599 | 0.344955288 |
| ENSG00000177494.5_2 | ZBED2 | protein_coding | -1.954299586 | 0.258046043 | 0.000437036 | 0.345786603 |
| ENSG00000163735.6_2 | CXCL5 | protein_coding | -1.890335597 | 0.269744304 | 0.013419396 | 0.832843697 |
| ENSG00000155657.26_3 | TTN | protein_coding | -1.802553682 | 0.286666717 | 0.008367975 | 0.807190972 |
| ENSG00000159713.10_2 | TPPP3 | protein_coding | -1.694750039 | 0.308908176 | 0.010752289 | 0.812222054 |
| ENSG00000179813.6_2 | FAM216B | protein_coding | -1.678323045 | 0.312445606 | 0.001272233 | 0.434723872 |
| ENSG00000163492.14_3 | CCDC141 | protein_coding | -1.629745432 | 0.323145223 | 0.027391728 | 0.905212674 |
| ENSG00000128536.15_3 | CDHR3 | protein_coding | -1.532495185 | 0.345678987 | 0.00147587 | 0.437833793 |
| ENSG00000160401.14_3 | CFAP157 | protein_coding | -1.461473268 | 0.363122123 | 0.00018081 | 0.344955288 |
